# LA-ICP-TOFMS Imaging Reveals Significant Influence of Cancer Cell Resistance on Oxaliplatin Compartmentalization in the Tumor Microenvironment

**DOI:** 10.1021/jacsau.5c00217

**Published:** 2025-06-11

**Authors:** Martin Schaier, Dina Baier, Sarah Theiner, Walter Berger, Gunda Koellensperger

**Affiliations:** 1 Institute of Analytical Chemistry, Faculty of Chemistry, 27258 University of Vienna, Vienna 1090, Austria; 2 Institute of Inorganic Chemistry, Faculty of Chemistry, 27258 University of Vienna, Vienna 1090, Austria; 3 Center for Cancer Research and Comprehensive Cancer Center, 27271 Medical University of Vienna, Vienna 1090, Austria

**Keywords:** mass spectrometry, laser ablation, antitumor agents, bioimaging, single-cell analysis, chemoresistance

## Abstract

Chemoresistance in cancer cells, particularly in refractory types, such as colorectal cancer, poses a major challenge to effective treatment. In particular, the interaction between cancer cells and the tumor microenvironment (TME) has been shown to exert substantial influence on the efficacy of therapeutic agents. This study investigated whether an intrinsic resistance phenotype alters drug distribution in the TME using xenograft models derived from HCT116 colorectal cancer cells, including oxaliplatin (OxPt)-sensitive and OxPt-resistant (OxR) variants. Tumors were prepared as formalin-fixed paraffin-embedded (FFPE) sections, followed by single-cell analysis with laser ablation inductively coupled plasma time-of-flight mass spectrometry (LA-ICP-TOFMS). Based on histological evaluations, a panel of metal-conjugated antibodies was designed to target tissue architecture and distinct cell states within the TME. A dedicated calibration strategy was applied to accurately measure platinum (Pt) uptake in phenotypically defined single cells across both the tumor and its microenvironment. The results revealed substantial structural differences: HCT116/OxR tumors exhibited robust growth following drug administration, while parental tumors displayed extensive degradation. Notably, OxPt accumulated significantly in necrotic regions specific to HCT116/OxR, indicating resistance-dependent changes in drug compartmentalization. These findings suggest that an intrinsically resistant cancer cell phenotype is capable of markedly altering metal distributions within the TME.

## Graphical abstract

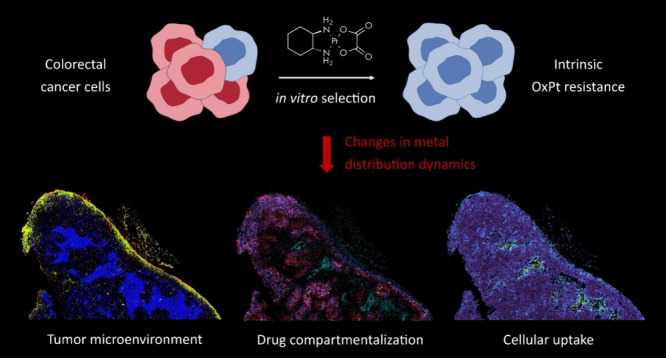

## Introduction

Colorectal cancer (CRC) is one of the most prevalent forms of cancer worldwide, with over 1.5 million new cases diagnosed annually.^1–3^ The standard treatment for this disease typically involves a combination of surgical intervention, radiotherapy, and OxPt- or irinotecan-based chemotherapy.^4–10^ In the last few decades, there has been a notable shift in the use of chemotherapy. Traditionally reserved for patients with moderate- to high-risk disease in the postoperative adjuvant setting, OxPt-based chemotherapy regimens are now being used preoperatively in the neoadjuvant setting, even in CRC patients with locally advanced, nonmetastatic disease.^11–13^ The neoadjuvant approach has been shown to significantly reduce the number of incomplete resections, resulting in improved disease control rates.^12,13^ Clinical studies are ongoing to evaluate the efficacy of adjuvant chemotherapy in patients who have received preoperative chemoradiotherapy.^14^

Despite these advancements, OxPt resistance, both intrinsic and acquired, remains a significant clinical challenge for CRC therapy and a crucial area of research. OxPt acts through DNA cross-linking, which disrupts replicative and transcriptional processes, ultimately leading to cancer cell death.^15–17^ However, resistance mechanisms are complex and multifaceted, involving the promotion of DNA repair via nucleotide excision repair (NER) and homologous recombination (HR), reduced drug accumulation, increased detoxification activity, and the inactivation of cell death signaling pathways.^18–23^

While this cancer cell-centered perspective on resistance development against Pt drugs has been the primary research focus for years, a holistic understanding of acquired and intrinsic resistance requires consideration of the tumor microenvironment (TME).^24^ Within the TME, physical and biological interactions play a pivotal role, involving cancer cell crosstalk with the immune and stromal compartments as well as the extracellular matrix. Resistance can arise from impaired drug delivery, cell death inhibition, inactivation of the Pt drug, and promotion of cancer cell stemness.^25^ For example, the stiffness and elasticity of the extracellular matrix affect drug delivery. Additionally, interactions between cancer cells and the extracellular matrix can promote cell-adhesion-mediated drug resistance. Acidic milieu in the TME can induce the expression of multidrug transporters in tumor cells, reducing the intracellular Pt drug accumulation. Shear stress and hypoxia have been shown to increase the cancer cell stemness. Epithelial-mesenchymal transition (EMT), in which tumor cells acquire stem cell-like properties, is a major contributor to tumor survival and chemoresistance.^26–28^ The multifaceted cellular crosstalk in the TME also has significant implications for drug resistance. For instance, cancer-associated fibroblasts (CAFs) in the tumor stroma were discovered to confer Pt drug resistance by activating autophagy.^29–31^ Furthermore, the modulation of regulated cancer cell death by CAFs is another aspect with implications for drug resistance.^30,31^ Resistance development might be based on either selection of a preexisting therapy refractory clone or on adaptation to survive the cytotoxic mechanism exerted by the Pt drug.^32,33^

Additionally, it remains unclear whether resistance development *in vivo* begins with the appearance of resistant tumor cell clones that, in turn, impact the TME to support cancer cell survival or whether resistance acquisition at the cancer cell level is preceded by facilitating changes in the microenvironment.^25,34^ Consequently, we have addressed in this study whether cancer cells that solely differ in sensitivity or acquired resistance against OxPt might alter the drug distribution in a xenograft tumor after short-term treatment. To answer this question conclusively, not only is an adequate isogenic tumor model with sensitive and resistant subline necessary but also a method allowing spatial dissection of the xenograft tissue with high resolution. Today, LA-ICP-MS is an essential tool for metal-based drug development. (Pre)­clinical studies have demonstrated the value of quantitative elemental bioimaging in assessing the tissue distribution of metal-based anticancer drugs.^35^ However, so far, single-cell level information has rarely been reached.^36–39^ A recent clinical study on colon cancer integrated histological examination with LA-ICP-MS bioimaging on FFPE tumor sections stained with hematoxylin and eosin (H&E).^40^ This multimodal approach allowed for the characterization of areas of OxPt enrichment, despite the fact that the implemented LA-ICP-MS platform did not allow for multiplexing or single-cell resolution. Interestingly, OxPt was found to be enriched in tumor areas with fibroblasts and hypothesized to be involved in therapy resistance. Our analytical approach in this study leverages rapid, high-resolution imaging as enabled by LA-ICP-TOFMS and the toolbox of multiplexed immunohistochemistry (IHC) by metal-labeled antibodies. As novelty, a unique calibration approach enables quantification of metal exposures at the single-cell level within the TME. This cutting-edge single-cell analysis pipeline allows to image and quantify Pt drug accumulation in phenotypically characterized single cells of tumor sections.^41^

Using this novel toolbox, we specifically addressed the questions of whether xenograft tumors derived from HCT116 CRC cells or their subline with *in vitro* acquired OxPt resistance (HCT116/OxR) differ concerning the TME and the compartmentation of OxPt following short-term *in vivo* treatment. Besides characterizing the Pt distribution in particular cell compartments of the TME, we find an unexpected but distinct relocation of Pt to necrotic tumor areas specifically in the xenograft tumors derived from the HCT116/OxR model. This indicates that resistant cancer cells are able to reshape the TME to support therapy failure.

## Results and Discussion

### Histological Investigations Reveal Distinct Microenvironmental Differences upon Resistance Development and Drug Treatment

Acquired OxPt resistance in the HCT116/OxR model, established in our lab by stepwise *in vitro* selection against OxPt, is mediated by a Pt accumulation defect.^42,43^ Underlying seems to be an efflux mechanism, which is increasing with exposure time and works in a glutathione-related manner.^44^ This resistance phenotype of HCT116/OxR as compared to the parental HCT116 cells and its impact on *in vivo* treatment response were compared by *in vitro* viability and *in vivo* treatment experiments, respectively (Figure S1). The distinct insensitivity of HCT116/OxR cells translated into a complete unresponsiveness of the respective xenograft *in vivo*, while growth of the parental HCT116 xenograft was gradually reduced by progressing OxR treatment up to more than 50% compared to the solvent-treated control.

Histological analysis of H&E-stained sections of untreated tumors revealed a comparable histological profile of both HCT116 xenograft tumors, indicating that the prior OxPt resistance selection *in vitro* did not significantly impact tumor histology (Figure S2). Generally, all tumors were characterized by the presence of viable tumor nodules surrounded by extensive tumor necrosis. Upon closer inspection, differences in the necrotic areas and perinecrotic regions were observed. The parental HCT116 model exhibited extensive necrotic areas with few intact nuclei and, likely, a low number of viable cells, while the HCT116/OxR tumors had a higher nuclear and cell density despite a similar extent of the necrotic core. Furthermore, the boundary between viable and necrotic cells was more distinct in the parental tumor with a dense layer of small, hyperchromatic nuclei encircling the living cell clusters.

Immunohistochemical staining for Ki-67 indicated the presence of similar proliferative regions, which were predominantly observed at the peripheries of both tumor types (Figures S3 and S4). However, as evident in the H&E stain, the parental HCT116 xenograft tumors displayed a more distinct demarcation of Ki-67-indicated cell proliferation between viable and necrotic areas. In contrast, Ki-67-positive cell nuclei were present throughout the (pre)­necrotic areas of HCT116/OxR xenografts. DNA damage assessment using the pH2AX marker showed a focus on proliferative areas in both tumors, but the distribution was more dispersed in the resistant tumor, particularly at the cell cluster peripheries, whereas the sensitive tumor exhibited a more uniform pattern. We determined CD44, a complexly spliced transmembrane glycoprotein and oncogene, as a marker for tumor aggressiveness and stemness.^45,46^ Both HCT116 xenograft models uniformly expressed CD44 on the cancer cells’ plasma membranes. Additionally, an unexpected and substantial deposition of this protein was observed within the necrotic regions of both tumor types. Vimentin staining revealed the presence of a fibroblastoid capsule and regular fibroblast layers in both tumor types. Moreover, endothelial cells of microvessels stained weakly positive for vimentin, in accordance with the literature.^47,48^ The sensitive parental tumors exhibited a more pronounced microvasculature, indicating enhanced stromal interaction and angiogenic support.^49^ These findings suggest that intrinsic resistance in HCT116 tumors may result in a more heterogeneous necrotic pattern characterized by resilient cell populations with enhanced tolerance to drug treatment.

Short-term analysis 72 h after a single-dose OxPt treatment on smaller xenograft tumors (see Figure S5) revealed alterations in the tissue structure of the parental tumor, indicative of a therapeutic response. Bright field microscopy, H&E staining, and IHC showed a high prevalence of microcavities and irregularities within the tumor stroma of the parental tumor model. A small area of necrosis was observed, with numerous cells displaying proliferative activity, albeit at a lower level than that observed in the OxPt-resistant tumor. Additionally, a high density of microvessels was noted throughout the sensitive tumor. In contrast, the HCT116/OxR tumor exhibited more extensive areas of necrosis surrounded by distinct zones of proliferating cells with lower vascularization.

### Multiplexed Imaging Mass Cytometry Confirms Histology and Expands the Phenotypic Characterization

Imaging mass cytometry by LA-ICP-TOFMS was used to identify distinct phenotypes revealed upon OxPt treatment. The metal-labeled antibody panel selection (Table S1), based on the histological evaluation, characterized the tumor’s structure, growth, and necrosis together with spatial immune cell and stromal cell localizations. The markers alpha-SMA (myofibroblasts), vimentin (mesenchymal cells and fibroblasts), pan-keratin (epithelial intermediate filaments), and collagen type I (extracellular matrix) specifically targeted the tumor stroma (Figure 1). The extent of multiplexing achieved by LA-ICP-TOF-MS analysis is shown in Figures S6 and S7, depicting the tumors, as imaged by the complete marker panel. Despite OxPt treatment, the HCT116/OxR tumor exhibited an organized tissue structure defined by the presence of CAFs and a dense collagen layer in the outer regions, acting as a physical barrier (Figure 1).

**Figure 1:**
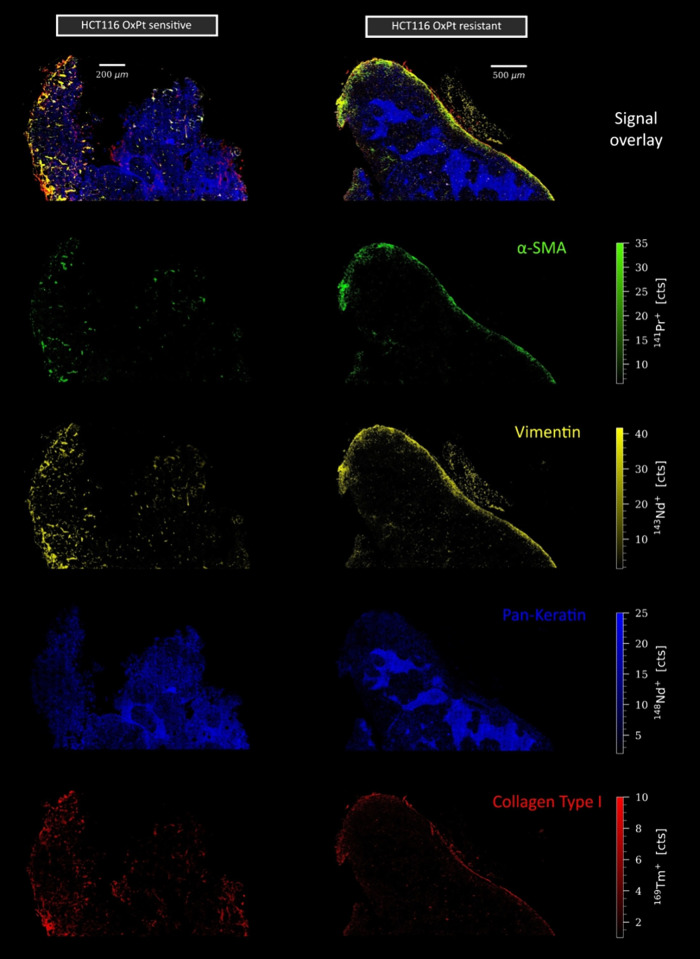
Structural comparison between parental and resistant HCT116 tumors after OxPt treatment. To ascertain the state of the tumor stroma, the markers alpha-SMA, vimentin, pan-keratin, and collagen type I were employed. LA-ICP-TOFMS analysis was performed at a 300 Hz acquisition rate with a 1 μm pixel size.

These structural characteristics were similar to those observed in untreated tumors (Figure S8). However, the presence of minimal collagen within the tumor itself indicates a potential limitation in endothelial support. Notably, the majority of epithelial cancer cells demonstrated keratin expression with a significant increase in areas of necrosis. CD44 demonstrated a comparable pattern (Figure S6), with extensive accumulation evident within regions of necrosis, which is consistent with the findings of the histological analysis. In contrast, the parental tumor appeared more disorganized upon drug treatment, with a lower expression of pan-keratin and CD44 (Figure S7). However, we observed higher collagen deposition in the intratumoral regions surrounding the epithelial cells (Figure 1).

The extended mass range of the developed LA-ICP-TOF-MS approach allows for simultaneous imaging of iron (Fe). It has been shown that, despite the use of staining procedures, this endogenous element retained its tissue distribution.^50^ Figure 2 illustrates how short-term OxPt exposure affected the Fe distribution of the sensitive as compared to the resistant xenograft model. The HCT116/OxR tumor exhibited low and irregular vascularization with large, clearly defined regions of ischemia, similar to untreated tumors. These features are typically indicative of a rapid and aggressive tumor growth. In contrast, the parent tumor showed significantly higher amounts of Fe distributed throughout with numerous visible microvessels. This finding aligns with the histological observations depicted in Figure S5. The augmented iron content could be indicative of hemorrhage directly induced by OxPt treatment or as a consequence of altered tissue compactness resulting from the initiated treatment response. The aforementioned observations suggest the presence of distinctive structural characteristics in the HCT116/OxR tumor, which may potentially influence the efficacy of the administered drug. In particular, the integrity of the tumor stroma in HCT116/OxR suggests that the OxPt distribution and subsequent treatment response may be affected.

**Figure 2:**
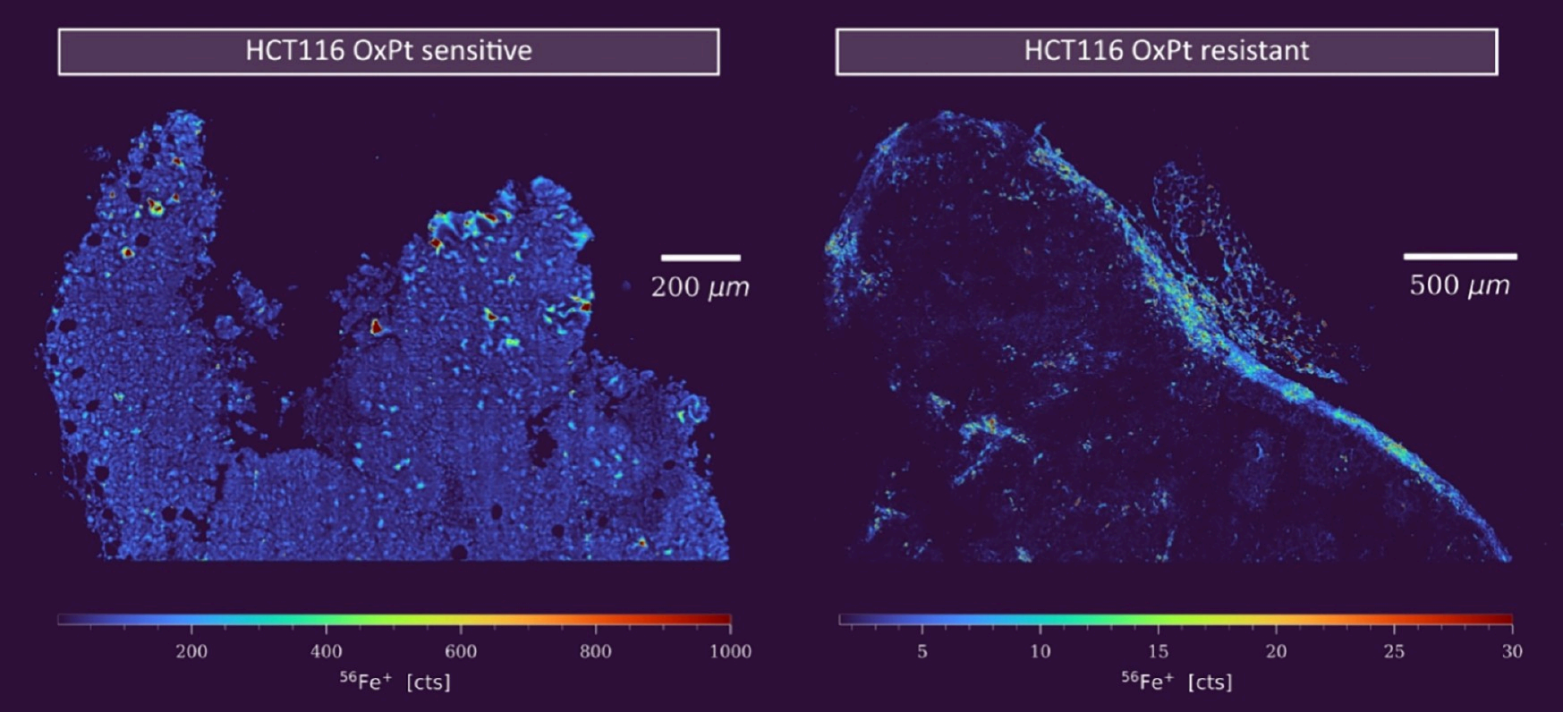
Fe signal intensity maps for both parental and resistant HCT116 tumors. LA-ICP-TOFMS analysis was performed at a 300 Hz acquisition rate with a 1 μm pixel size.

Due to the SCID background, HCT116 xenograft tumor models inherently exhibit a lack of B- and T-cells.^51,52^ As a consequence, the presence of both CD3- and CD19-positive cells was minimal (Figures S6 and S7). Notably, small populations of F4/80-positive macrophages, which were also positive for CD45 and CD11b, were identified within the tumor stroma.

### Quantification Strategy for OxPt at Single-Cell Level in Tissue is Fit-for-Purpose

In the next step, we focused on the Pt drug distribution measured simultaneously with the marker panel, leveraging the multiplexing capabilities of LA-ICP-TOF-MS. Previous research showed that metal compounds that form macromolecular complexes in tissue could be quantified, despite the extensive sample preparation necessitated by IHC.^41^ A tailored calibration strategy enables the quantitative bioimaging of metals in the (sub-)­fg per cell concentration range. However, validation experiments for each investigated metal species are needed to prove the method fit-for-purpose.^53,54^ OxPt is known to form complexes with blood or cellular macromolecules in tissues.^55,56^ Comparison of consecutive metal-labeled and unlabeled sections using k-means clustering demonstrated minimal washout effects of OxPt, with the Pt content and distribution being consistent between both sections (Figure 3A). This was further confirmed by employing the same methodology for different organ sections (Figure S9), proving that the method is fit-for-purpose.

**Figure 3:**
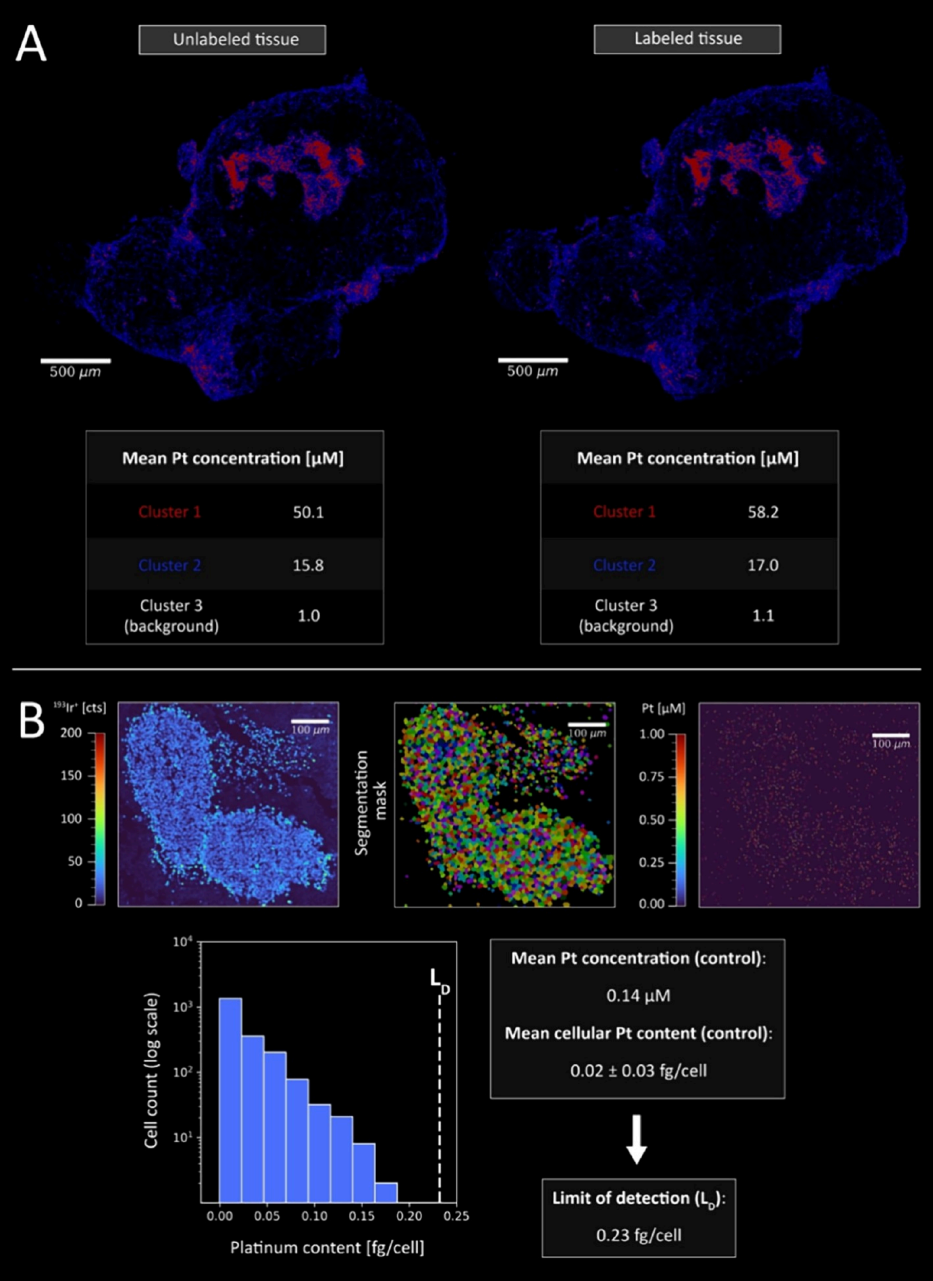
Validation of quantitative Pt bioimaging. (A) Pt distribution in consecutive tumor sections (HCT116/OxR), with and without immunolabeling. Pt concentrations were calculated using k-means clustering (*n* = 3), based on the pixel area and tissue thickness (5 μm), and reported in μM. (B) Cell nuclei, segmentation mask, and Pt distribution in an untreated HCT116 tumor (control). Minimal Pt background was detected (mean of 0.14 μM), with a histogram showing the distribution of measured Pt content per cell (mean of 0.02 ± 0.03 fg/cell). They were included to characterize the baseline Pt distribution in the absence of OxPt treatment. Based on this distribution, a tissue-specific limit of detection (L_D_) for Pt in tumor cells was defined as 0.23 fg/cell.

Pt quantification is conducted at either the pixel or the single-cell level. The latter approach necessitates the use of segmentation algorithms during image analysis. The identification of cellular objects is typically reliant upon the use of cell nuclei and membrane markers; however, alternative approaches have been demonstrated in recent publications, such as coregistration with histological images.^57^ The present study employed a combination of CD44 (cancer stem cells and mesenchymal cells) and E-cadherin (epithelial cells) to label cell membranes. This approach encompasses the majority of cells and accounts for the observed variability in expression levels across different tumor regions, as illustrated in Figure S10. Notably, there was a correlation between E-cadherin downregulation and CD44 overexpression. The nuclei were labeled with the established iridium (Ir) intercalator. Using the tailored MeXpose image analysis pipeline, quantitative single-cell Pt data were obtained. Segmented cells derived from LA-ICP-TOFMS imaging of control tumors were analyzed, revealing a compound Poisson distribution, from which a procedural detection limit (L_D_) of 0.23 fg/cell was established (Figure 3B).^58^ Despite the overall Pt background in the tissue being low (exhibiting a mean tissue concentration of 0.14 μM and a mean cellular content of 0.02 fg/cell), the observed variability resulted in a comparatively higher L_D_. Nevertheless, this threshold constitutes a stringent estimate, ensuring exclusion of background Pt values observed in control cells that are considered nonsignificant.

### Pixel-Based Image Analysis Identifies Substantial OxPt Deposits within the Tumor Stroma and Regions of Necrosis

The first step involved scrutinizing the Pt distribution in relation to marker expression using pixel-based image analysis. To elucidate Pt enrichment in actively proliferating tumor regions, the following markers were examined: Ki-67 (proliferation), pS6 (mTOR pathway activation and active protein synthesis), and pHistone H3 (mitosis). In both tumor types, it was clear that the majority of OxPt was localized outside the viable and proliferative tumor regions (Figure 4).

**Figure 4:**
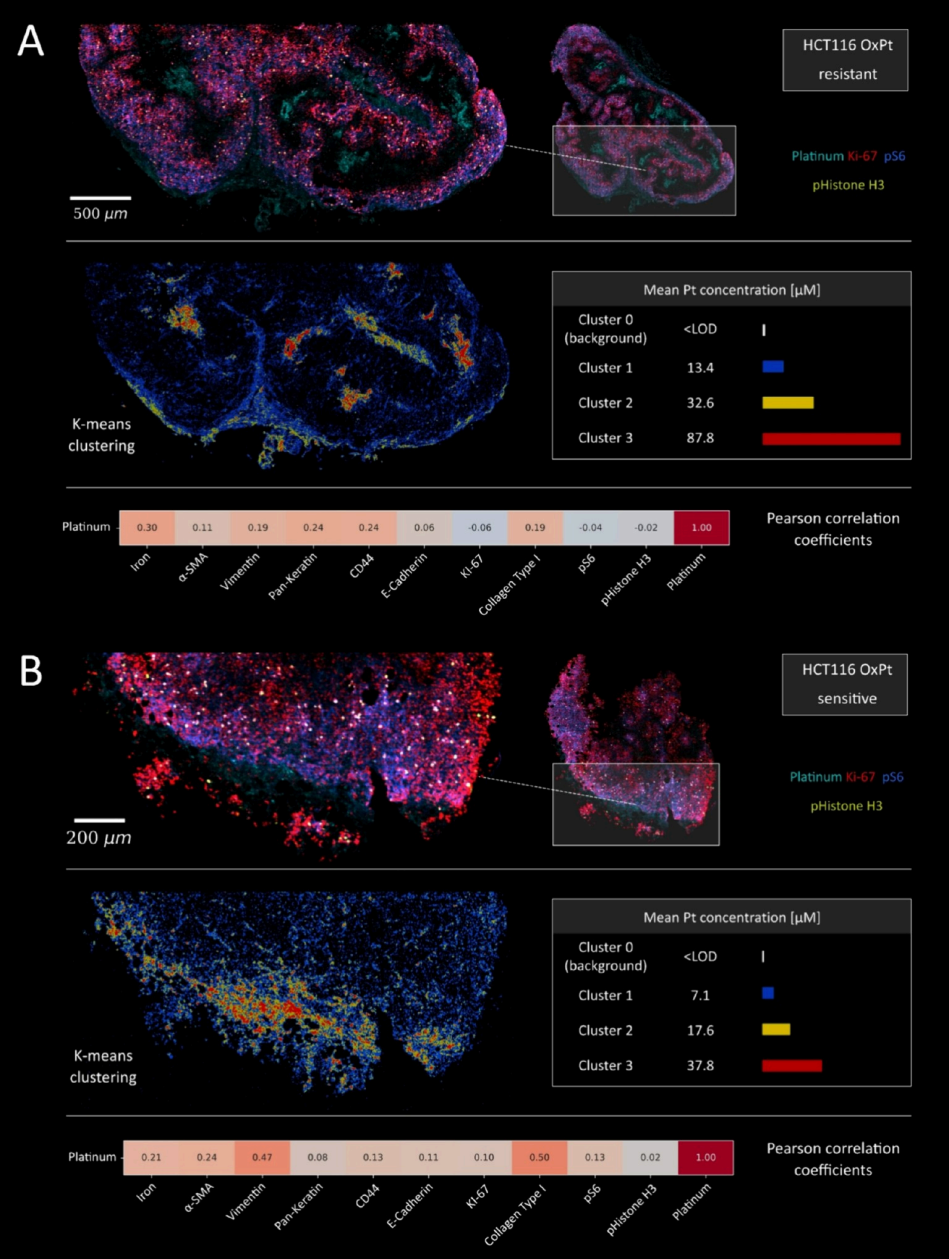
Comparative analysis of Pt deposition in (A) resistant and (B) parental HCT116 tumors. The markers Ki-67, pS6, and pHistone H3 highlight the structure of the active tumor parenchyma. Images were acquired by using LA-ICP-TOFMS at a repetition rate of 300 Hz and a pixel size of 2.5 μm. *K*-means clustering (*n* = 4) was applied to the Pt distributions, providing a clear representation of Pt accumulation and concentration within the tumor. Pearson's correlation was used to assess the colocalization and intensity overlap between Pt and a range of markers for both tumor stroma and parenchyma.

A more detailed insight into the deposition of Pt within tumors was gained by examining its distribution using k-means clustering. Surprisingly, Pt levels in the HCT116/OxR tumor were considerably higher than those observed in the parental tumor. The lowest Pt accumulation was observed within the tumor parenchyma, with an average value of 13.4 μM. Significant Pt deposits were observed in areas with CAFs and vascularized regions, averaging approximately 32.6 μM. Specifically in the HCT116/OxR tissue, the majority of OxPt was localized in the necrotic regions, exhibiting average concentrations of 87.8 μM, with some areas showing even higher Pt levels. This pattern was observed in multiple HCT116/OxR tumors (Figure S11). These results suggest that cancer cells harboring acquired OxPt resistance can directly or indirectly sequester Pt outside living cells, potentially evading cytotoxic effects.

Pearson’s correlation analysis revealed a negative correlation between Pt and tumor growth markers (Ki-67, pS6, and pHistone H3). In contrast, there was a positive correlation with tumor stroma markers (vimentin, α-SMA, iron, and collagen type I) and necrosis-associated markers such as pan-keratin and CD44, further supporting these findings. In the parental HCT116 tumor model, the lowest Pt levels were found in the active tumor regions, with a value of 7.1 μM. The tumor stroma showed significantly elevated Pt concentrations, with areas containing myofibroblasts exhibiting levels of 17.6 μM, while collagen-rich regions and fibroblasts had even higher Pt concentrations, reaching 37.8 μM. These observations are further supported by the Pearson correlation analysis, which revealed a strong positive correlation between Pt and both vimentin and collagen, with a weaker but still notable correlation to α-SMA. Tumor growth markers demonstrated a stronger correlation with Pt in the parental tumor compared to the HCT116/OxR model, although they remained at lower levels than those observed in the tumor stroma. In both tumors, the tumor stroma appears to function as an effective physical barrier to OxPt, limiting the drug’s access to the viable tumor cells. This is consistent with previous studies showing prolonged retention of OxPt in CAFs following cessation of treatment.^40,59^

### Phenotypic Screening Reveals Differential Cellular Uptake of OxPt in HCT116/OxR Tumors

Finally, quantitative Pt imaging was integrated with phenotypic screening at the single-cell level. Using segmented cellular data, unsupervised PhenoGraph clustering was applied, identifying distinct cellular phenotypes based on antigen expression, as depicted in the heatmap (Figure 5B). Figure S12 shows the spatial distribution of the cell clusters within the tumor tissue.

**Figure 5:**
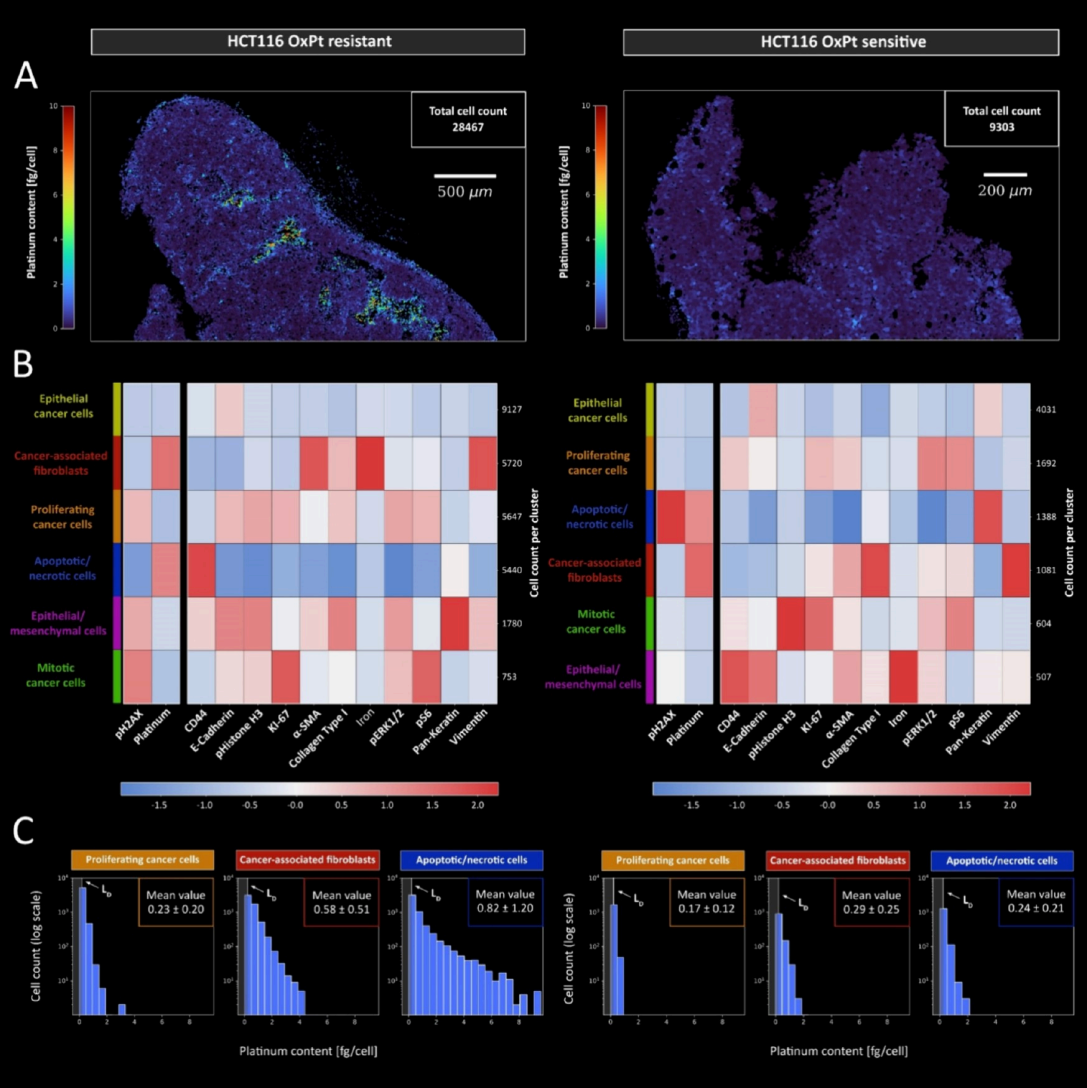
Cellular uptake of Pt in resistant and parental HCT116 tumors. (A) To visualize the cellular Pt content, the segmentation mask was overlaid with the Pt distribution. (B) PhenoGraph clustering was performed, and cellular phenotypes were assigned based on antigen expression. (C) Histograms of cellular Pt distribution are shown for proliferating cancer cells, CAFs, and apoptotic/necrotic cells, as these showed the most significant differences. Inclusion of the previously established detection limit in the histograms helped to highlight significant cellular OxPt accumulation.

Epithelial cancer cells were identified based on the presence of E-cadherin and pan-keratin, alongside low expression of other markers. Proliferating cancer cells exhibited elevated levels of Ki-67 and pS6, while cells undergoing active mitosis showed distinct histone H3 phosphorylation. Clear identification of CAFs was enabled through the presence of vimentin, α-SMA, collagen, and Fe. Cells classified as “apoptotic/necrotic” were characterized by an intact nucleus, elevated CD44, and pan-keratin expression, with significantly negative correlations to all other investigated markers. Cells exhibiting both epithelial and mesenchymal characteristics but lacking proliferative activity were categorized as epithelial/mesenchymal cells.

Single-cell analysis revealed that both tumors showed the highest Pt correlation with CAFs and apoptotic/necrotic cells, with the former being more pronounced in the parental tumor (Figure 5B). DNA damage of HCT116 xenografts was primarily concentrated in apoptotic/necrotic cells and exhibited a strong correlation with Pt accumulation, whereas in HCT116/OxR, DNA damage was more diffusely distributed in proliferating and mitotic cells. The DNA damage pattern in the HCT116/OxR tumor shared similarities with that of the untreated control. The lack of OxPt-related DNA damage may suggest either a reduced ability of OxPt to induce DNA damage or an increase in DNA repair mechanisms, both of which could contribute to enhanced cell survival. The Pt content in the parental tumor was relatively uniform across the majority of cells, with average amounts ranging from 0.2 to 0.3 fg/cell and maximum values reaching 2 fg/cell (Figure 5C). CAFs exhibited a notable increase in Pt accumulation with an average of 0.29 fg/cell. Although a significant proportion of the cells displayed Pt levels below the detection limit of 0.23 fg/cell (Figure 3), the majority of cells demonstrated Pt accumulation above this threshold. In the HCT116/OxR, Pt content in the parenchyma was similar to that in the parental tumor, with proliferating cancer cells averaging 0.23 fg/cell and some cells reaching up to 3 fg/cell. However, CAFs and apoptotic/necrotic cells in HCT116/OxR exhibited significantly higher Pt levels with averages of 0.58 and 0.82 fg/cell, respectively. Notably, some necrotic cells in the resistant tumor reached Pt levels as high as 10 fg/cell. These findings suggest that drug-resistant tumors may limit OxPt accumulation in epithelial cells, consistent with the results of pixel-based image analysis.

### Systemic OxPt Distribution Unveils Significant Accumulation in Spleen

To gain insight into both the accumulation of OxPt within tumors and its systemic distribution, a comprehensive analysis was conducted on vital organs, including the kidney, spleen, liver, and lung. The distribution and concentration of Pt within these organs were found to be nearly identical in mice bearing HCT116 and HCT116/OxR xenograft tumors. The discussion will therefore focus on the discrepancies among the different organs. Figure 6 provides an overview of Pt levels across these organs, showing that the liver, kidney, and lung had average Pt levels comparable to the tumor sections (Figure S13), ranging from 10 to 20 μM, indicating a relatively uniform distribution throughout the body.

**Figure 6:**
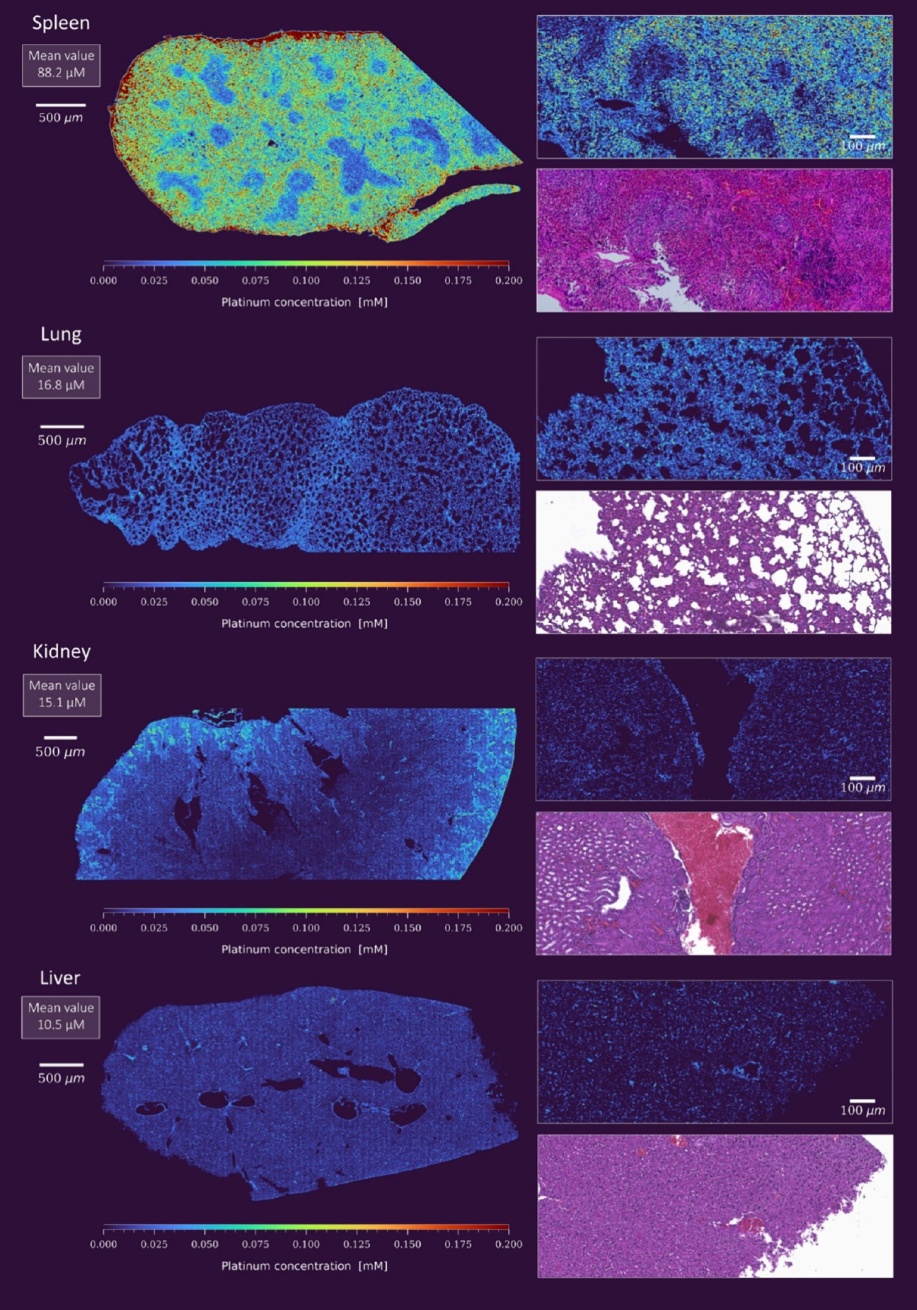
Quantitative Pt imaging in organ sections from an OxPt-treated mouse. The analysis was performed using LA-ICP-TOFMS imaging with a 300 Hz acquisition rate. Lower-resolution images (2.5 μm pixel size) provided an overview of Pt distribution across various organs (left). High-resolution close-up images (1 μm pixel size) allowed more detailed examination at the cellular level (right). Consecutive sections stained with H&E give an insight into the structural features of the organs where Pt accumulation has taken place.

However, localized Pt accumulations were observed in selected tissues, as seen in Figure S14. In the liver, elevated Pt concentrations, up to 50 μM, were found primarily in the endothelial cells of arteries and veins, particularly in the portal triad. In the kidney, the tubules and blood vessels of the cortex exhibited heightened values, with the greatest concentration observed in the renal capsule, reaching a maximum of approximately 100 μM. Similar concentration levels were detected in the pulmonary capillaries of the lung, especially those enriched with erythrocytes. In contrast, the spleen showed significantly higher Pt concentrations, averaging 88.2 μM and reaching as high as 1000 μM, predominantly localized within the red pulp and capsule, suggesting a tendency for Pt to accumulate in highly vascularized regions. Further analysis revealed a positive Pearson correlation coefficient between Fe and Pt levels across all organs with a particularly strong correlation in the spleen, as shown in Figure S15. The spleen also demonstrated significantly elevated Fe levels, surpassing those in other organs by more than 10-fold. Histologic examination using H&E staining revealed a less distinct border between red and white pulp compared to typical histologic profiles, as well as a pronounced prevalence of siderophages. These findings suggest an increased turnover of blood cells within the spleen, potentially contributing to OxPt-induced anemia.

Additionally, significant DNA damage was observed within the splenic parenchyma, as illustrated in Figure S16, supporting this hypothesis. This heightened blood turnover likely underlies the mechanism driving splenomegaly, characterized by a substantial increase in spleen volume, a phenomenon observed in nearly 90% of patients undergoing OxPt treatment.^60,61^ The intricate interplay among OxPt exposure, blood turnover dynamics, and resultant spleen enlargement underscores the multifaceted physiological repercussions of OxPt therapy and warrants further investigation into its hematotoxic effects.

## Conclusions

In this study, we show the potential of LA-ICP-TOFMS imaging to elucidate the distribution of anticancer metal drugs, in the current case, OxPt, in cancer tissue at the single-cell level. A comprehensive picture of the distribution of not only the therapeutic metal but also endogenous metals such as Fe in cancer cells as compared to cells of the microenvironment was generated. Specifically, we have addressed the question of whether a cancer cell intrinsic resistance phenotype, established by *in vitro* selection against OxPt, can impact the metal drug distribution not only in cancer cells but also within microenvironmental compartments. Interestingly, we found massive Pt deposition within defined parts of necrotic areas in HCT116/OxR, but not in parental HCT116 xenograft tumors. This drug deposition phenomenon was not based on general differences in the prevalence of necrotic areas between the sensitive and resistant models but was mediated by different drug compartmentalization events based on crosstalk between cancer cells and microenvironmental components. OxPt-accumulating necrotic regions were characterized by the presence of strongly hematoxylin-positive small nuclei, remnants of massive innate immune infiltration, but they lacked signs of any cell viability or proliferation. Pt localization was detected outside of these cell remnants, suggesting rather active Pt deposition in the extracellular space by cancer cells than the accumulation of dying immune cells with high Pt accumulation. In summary, we prove by the use of LA-ICP-TOFMS that a cancer cell intrinsic resistance phenotype can massively impact metal distribution dynamics in the cancer microenvironmental space. Comparable analyses in surgical specimens of human CRC patients after neoadjuvant OxPt-based treatment are warranted to elucidate if Pt accumulation in necrotic areas might predict therapy failure under clinical conditions.

## Methods

### Chemicals and Reagents

The ELGA water purification system (Purelab Ultra MK 2, United Kingdom) was used to provide ultrapure water with a resistivity of 18.2 MΩ cm for all dilutions and washing procedures. The multielement stock solution was obtained from Labkings (Hilversum, The Netherlands). Lyophilized bovine serum albumin (BioReagent), BioUltra-grade Tris-buffered saline, anhydrous m-xylene (purity ≥ 99%), and EMSURE absolute ethanol, along with cell culture media and reagents, were sourced from Sigma-Aldrich (Steinheim, Germany). Thermo Fischer Scientific (Waltham, MA, USA) provided the Tween-20 detergent solution (Surfact-Amps, 10%) and SuperBlock blocking buffer in TBS. Target retrieval solution at pH 9, containing Tris/EDTA was supplied by Agilent Technologies (Waldbronn, Germany). The metal-labeled antibodies (Table S1) and Ir intercalator (Cell-IDTM, 125 μM) were purchased from Standard BioTools (San Francisco, CA, USA). The CD16/32 antibody was obtained from BD Biosciences (San Jose, CA, USA). Plastic dishes, plates, and flasks were procured from StarLab (Hamburg, Germany).

### Cell Culture

Dr. Vogelstein of Johns Hopkins University (Baltimore, USA) kindly provided the HCT116 human CRC cell line. The subline HCT116/OxR was selected for acquired oxaliplatin (OxR) resistance as published.^44^ Cells were grown in McCoy’s medium (Sigma-Aldrich, St. Louis, MO, USA) supplemented with 10% fetal calf serum (PAA, Linz, Austria) and 2 mM glutamine (Sigma-Aldrich, St. Louis, MO, USA). Cultures were kept in standard cell culture conditions and regularly checked for Mycoplasma contamination.

### Cell Viability Assay *In Vitro*

To determine the impact of OxPt treatment on cell viability, 3 × 10^3^ HCT116 or HCT116/OxR cells were seeded in 96-well plates in 100 μL of McCoy’s medium and allowed to recover for 24 h. Subsequently, cells were treated with the respective OxPt concentrations, prepared in an additional 100 μL of medium, for a 72 h continuous drug exposure. Cell viability was measured by an MTT-based survival assay (EZ4U; Biomedica, Vienna) as described previously.^62^

### Animal Experiments

*In vivo* experiments were conducted by injecting 1 × 10^6^ HCT116 or HCT116/OxR cells subcutaneously into the right flank of 11-week-old male CB-17/SCID mice using serum-free RPMI medium as solvent (Sigma-Aldrich, St. Louis, MO, USA). Animal experiments were performed in accordance with the regulations of the Ethics Committee for the Care and Use of Laboratory Animals at the Medical University of Vienna (application number BMWF-66.009/0140-II/3*b*/2011), the US Public Health Service Policy on Human Care and Use of Laboratory Animals, and the United Kingdom Coordinating Committee on Cancer Prevention Research Guidelines for the Welfare of Animals in Experimental Neoplasia. The animals were housed in a pathogen-free environment within a laminar airflow cabinet. Tumors were palpable on day 7 following the injection. The animals’ condition was observed on a daily basis in order to identify any indications of distress. Furthermore, the size of the tumors was evaluated at regular intervals through the use of a caliper measurement. Tumor volume was calculated according to the formula (length × width^2^/2). For activity determination, tumor-carrying animals (*n* = 4) were treated intraperitoneally twice a week for 2 weeks with 9 mg kg^–1^ OxPt or physiological NaCl solution with 5% glucose as solvent control. Mean tumor volumes were calculated, and the ratio between OxPt- and solvent-treated controls was evaluated. For imaging experiments, the animals were intraperitoneally treated with a single dose of OxPt. Mice were euthanized 72 h after treatment. This time point was chosen to avoid the presence of higher amounts of Pt within the blood vessels. The tumors and organs were fixed for 24 h in a 4% formaldehyde solution (Carl Roth) and then paraffin-embedded using a KOS machine (Milestone Medical, Sorisole, Italy).

### Histological Analysis

For the IHC, sections of paraffin-embedded tumors and organs were prepared, deparaffinized, and rehydrated. Endogenous peroxidases were blocked using H_2_O_2_-containing PBS. Tissue sections on slides were boiled in 10 mM citrate buffer, and nonspecific binding sites were blocked with UltraVision LP blocking reagent according to the manufacturer’s instructions (Thermo Fisher Scientific). Sections were then incubated with the following antibodies: pH2AX (clone JBW301, Sigma-Aldrich; 1:500, for 90 min at room temperature (RT)), Ki67 (clone MIB-1, Dako; 1:100, for 90 min at RT), vimentin (clone D21H3, Cell Signaling; 1:500, for 90 min at RT), and CD44 (clone EZK2Y, Cell Signaling; 1:300, for 90 min at RT). Primary antibody binding was visualized using the UltraVision LP detection system as recommended by the manufacturer (Thermo Fisher Scientific) and developed with 3,3′-diaminobenzidine (Dako). Sections were counterstained with hematoxylin Gill III (Merck). Additionally, H&E staining was performed.

### Immunolabeling with Metal-Conjugated Antibodies

After deparaffinizing the tissue sections in fresh xylene for 20 min, they were rehydrated through a graded ethanol series (100 to 70%) and subsequently washed with ultrapure water. Heat-mediated antigen retrieval was performed at 96 °C for 30 min with Tris-EDTA buffer at pH 9. The slides were allowed to cool, then washed again with ultrapure water and TBS/0.05% Tween. SuperBlock buffer was applied for 30 min at RT followed by CD16/32 treatment for 10 min to reduce the level of nonspecific binding. Following this, the sections were incubated overnight in a hydration chamber at 4 °C with a cocktail of metal-labeled antibodies (details in Table S1). The antibodies, diluted 1:50 in a solution containing 0.5% BSA, 1:100 CD16/32, and TBS/0.05% Tween, were centrifuged at 13,000 g for 2 min before use to prevent aggregation. The tissue sections were then labeled with an Ir intercalator (125 μM) at a 1:100 dilution in TBS/0.05% Tween. After a 5 min incubation at RT in the hydration chamber, the sections were thoroughly washed with ultrapure water and allowed to air-dry. Microscopic images were captured to provide an overview of the tissue sections before LA-ICP-TOFMS analysis.

### Calibration Strategy for LA-ICP-TOFMS Analysis

Schweikert et al. previously described the use of gelatin microdroplets for quantitative analysis of multiple elements within biological samples by LA-ICP-TOFMS.^53,54^ Commercial multielement standard solutions were mixed with gelatin and dispensed into 384-well plates. Using the CellenONE X1 microspotter (Cellenion, Lyon, France), arrays of microdroplet standards were created on glass slides. These droplets, approximately 200 μm in diameter and 400 ± 10 pL in volume, were analyzed by the instrument software, which assessed their size for normalization and the precise calculation of element quantities within the droplets. Whole microdroplets were subjected to quantitative ablation, followed by multielement analysis using LA-ICP-TOFMS.

### LA-ICP-TOFMS Analysis

Elemental imaging was performed using the Iridia 193 nm LA system from Teledyne Photon Machines (Bozeman, MT, USA), paired with the icpTOF 2R ICP-TOFMS instrument from TOFWERK AG (Thun, Switzerland). An aerosol rapid introduction system (ARIS) connected the ultrafast, low-dispersion LA cell within the cobalt ablation chamber to the ICP-TOFMS system. Optimal tuning conditions were achieved by introducing an argon makeup gas stream into the carrier gas stream prior to plasma entry, focusing on specific ion intensities, minimal oxide formation, and low elemental fractionation. Sampling was performed at a repetition rate of 300 Hz. Spot sizes ranged from 2 to 10 μm with interspacing between 1 and 5 μm and a fixed dosage of 2, providing a 2x overlap in both the *x* and *y* directions to optimize ablation. This facilitated both single-cell analysis and comprehensive tissue overviews. Samples, including microdroplets and various tissues, were completely ablated using optimized energy densities between 0.4 and 1.4 J cm^–2^ that ensured removal of all material without damaging the glass substrate, enabling reliable quantitative analysis. The icpTOF 2R ICP-TOFMS instrument had a specified mass resolution of 6000 and detected ions in the *m*/*z* range of 14 to 256. Detailed instrument parameters are listed in Table S2.

### Data Acquisition and Processing

LA-ICP-TOFMS data were acquired using TofPilot 2.10.3.0 from TOFWERK AG, with subsequent storage in hierarchical data format (HDF5). HDIP version 1.8.5.171 from Teledyne Photon Machines was used for additional data processing with an automated script generating two-dimensional elemental distribution maps. The processed data was exported in two formats: TIFF files for single-cell analysis and CSV files for pixel-based image analysis. The data was evaluated using the MeXpose image analysis pipeline, as described by Braun, Schaier et al.^41^ This entailed preprocessing, cell segmentation, data extraction, and downstream statistical analysis.

## Supplementary Material



## Data Availability

The data supporting the findings of this study are provided in the Supporting Information accompanying this article. Upon reasonable request, raw images of the data will be made available. For further inquiries, please contact Gunda Koellensperger at gunda.koellensperger@univie.ac.at.

## Notes

OxPt resistance confirmation; H&E stain of control tumors; IHC stain for control tumors; histological evaluation of OxPt-treated tumors; Signal intensity maps for each metal-conjugated antibody used on the OxPt-treated tumors; structural characterization of control tumor via metal-conjugated antibodies; evaluation of OxPt washout after staining in different organ tissues; cell segmentation strategy for tumors; OxPt distribution in biological replicates of HCT116/OxR tumor; visualization of cellular phenotypes in tumor tissues; OxPt concentration in tumor compared to organs; evaluation of Pt distribution in OxPt-treated organs; Pearson's correlation of OxPt with iron in OxPt-treated organs; DNA damage in OxPt-treated spleen; list of metal-conjugated antibodies; LA-ICP-TOFMS parameters (PDF)
