# Supplementary material for: LA-ICP-TOFMS Imaging Reveals Significant Influence of Cancer Cell Resistance on Oxaliplatin Compartmentalization in the Tumor Microenvironment
Source: JACS Au. 2025 Jun 11;5(6):2619–31. doi: 10.1021/jacsau.5c00217 (PMC12188412; doi:10.1021/jacsau.5c00217)
Supplement: Supplementary file 1 [file au5c00217_si_001.pdf]

Supporting Information

LA-ICP-TOFMS Imaging Reveals Significant Influence of Cancer Cell Resistance on Oxaliplatin Compartmentalization in the Tumor Microenvironment

Martin Schaier[1], Dina Baier[2,3], Sarah Theiner[1], Walter Berger[3]*, Gunda Koellensperger[1]*

[1] Institute of Analytical Chemistry, Faculty of Chemistry, University of Vienna, 1090 Vienna, Austria

[2] Institute of Inorganic Chemistry, Faculty of Chemistry, University of Vienna, 1090 Vienna, Austria

[3] Center for Cancer Research and Comprehensive Cancer Center, Medical University of Vienna, 1090 Vienna, Austria

* Corresponding authors:

Walter Berger
Center for Cancer Research and Comprehensive Cancer Center, 1090 Vienna, Austria
Tel: +43-1-40160-57555, Email: walter.berger@meduniwien.ac.at

Gunda Koellensperger
Institute of Analytical Chemistry, 1090 Vienna, Austria
Tel: +43-1-4277-52303, Email: gunda.koellensperger@univie.ac.at

Supplementary Figures S1 – S16

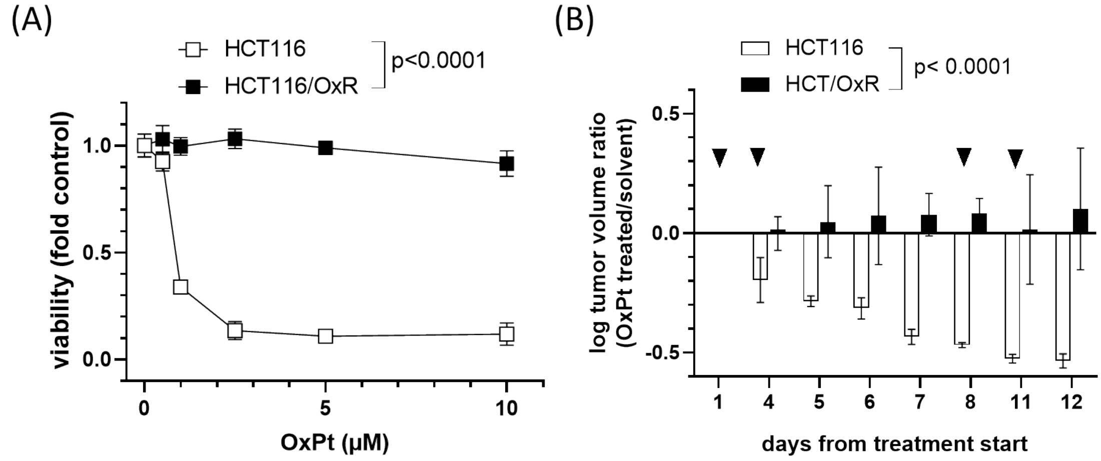

Figure S1: Confirmation of the acquired OxPt resistance phenotype in the HCT116/OxR model *in vitro* and *in vivo*. (A) Viability of HCT116 and HCT116/OxR cells after continuous OxPt exposure at the indicated concentrations for 72 h. (B) Mice (n = 4 per experimental group) bearing subcutaneous HCT116 or HCT116/OxR xenografts were subjected to OxPt or solvent therapy for two weeks at the time points indicated by arrowheads. Tumor volumes were calculated from caliper measurements. Data given are the log(10) ratio of OxPt-treated versus solvent-treated mean tumor volumes (mean and standard deviation) at the indicated time points. Differences were tested for significance by two-way ANOVA.

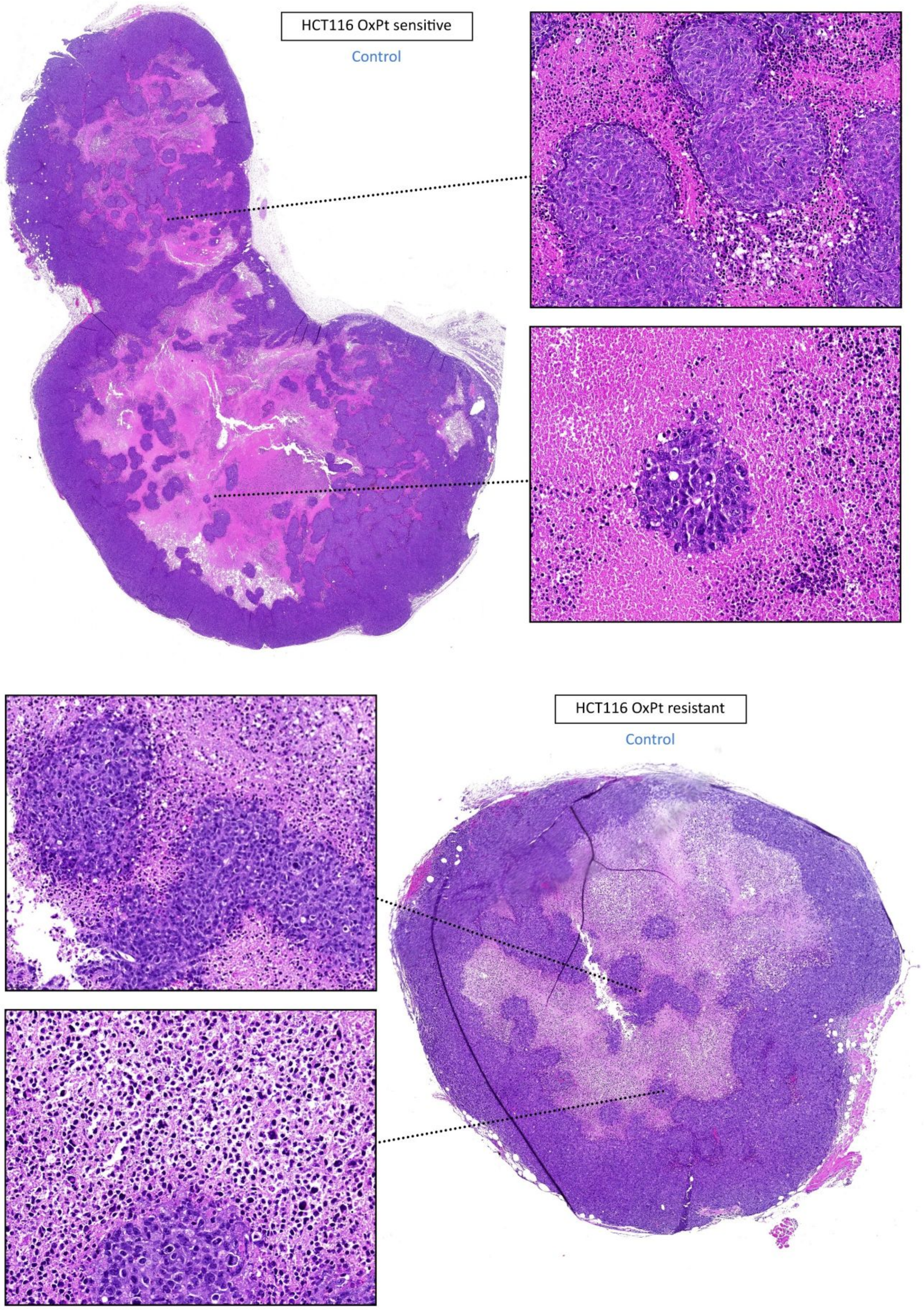

Figure S2: Microscopic images of parental and resistant HCT116 tumors without OxPt treatment after H&E staining. The close-up images provide a detailed view of the different types of tumor necrosis.

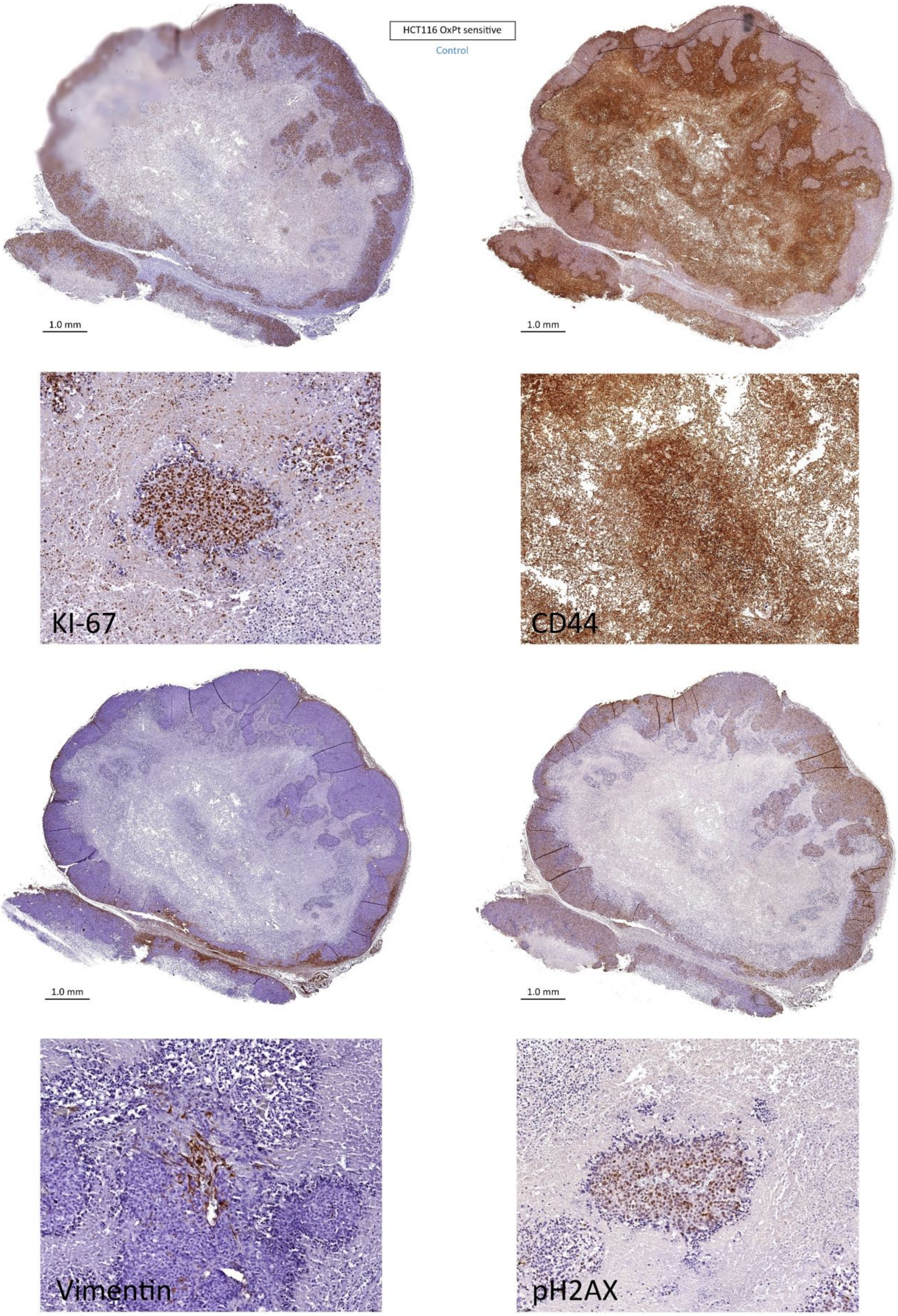

Figure S3: Microscopic images of the parental HCT116 tumor in the absence of oxaliplatin treatment. Consecutive tissue sections were labeled with antibodies and subjected to DAB staining. The close-up images provide a detailed view of specific regions of interest.

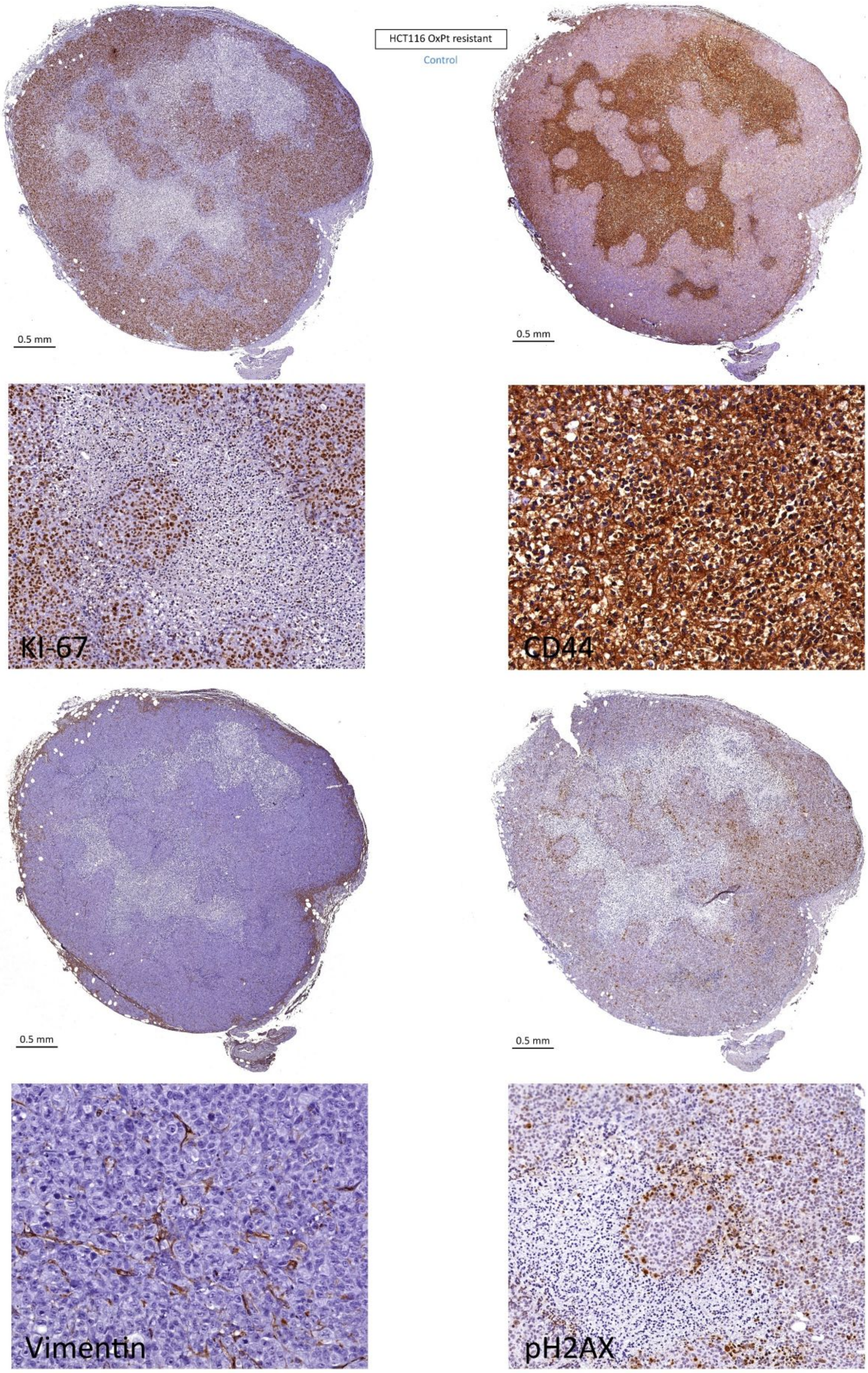

Figure S4: Microscopic images of the resistant HCT116 tumor in the absence of OxPt treatment. Consecutive tissue sections were labeled with antibodies and subjected to DAB staining. The close-up images provide a detailed view of specific regions of interest.

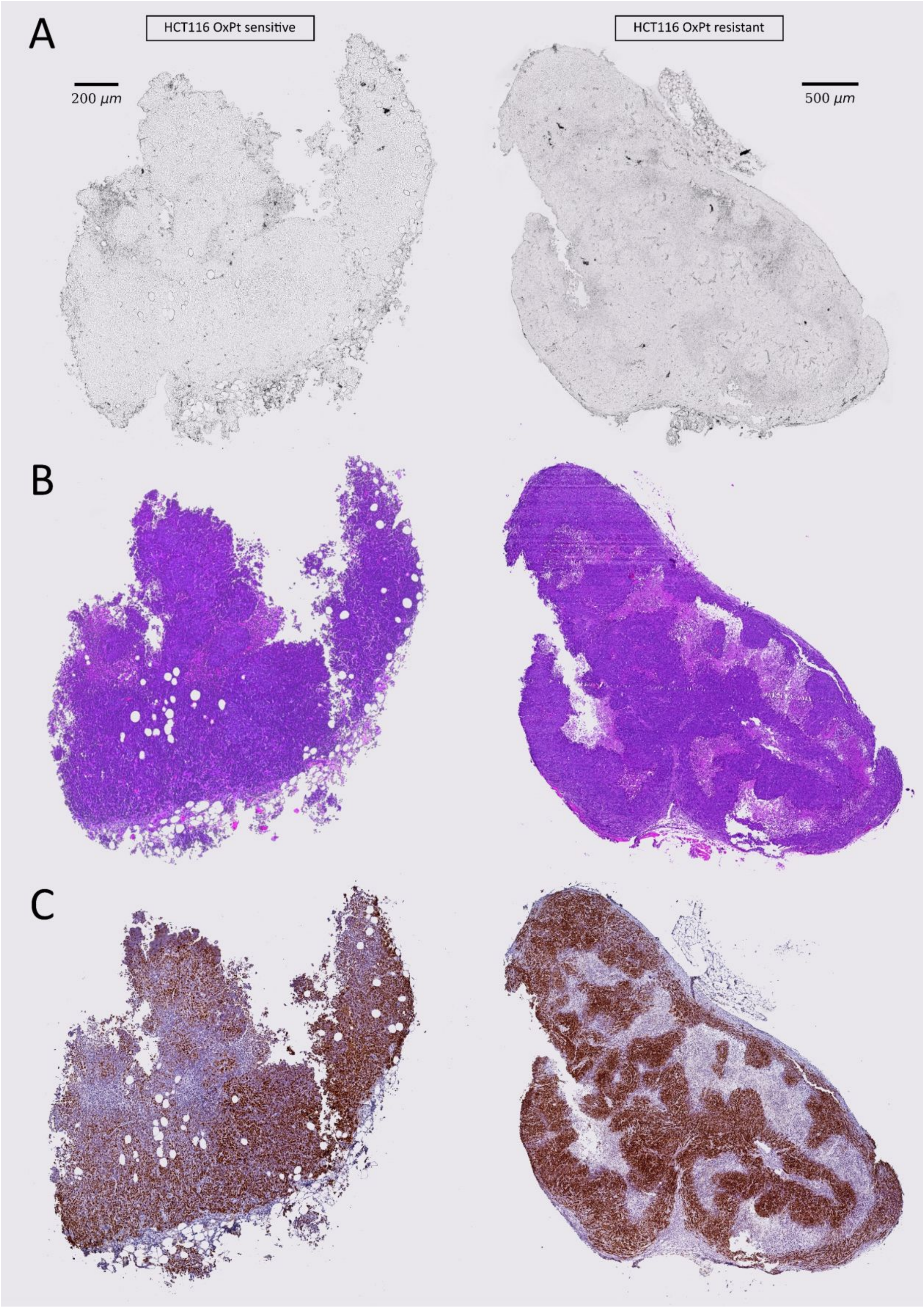

Figure S5: A comparison of the tissue structure between the resistant and sensitive tumors treated with OxPt was conducted using microscopic images. (A) shows a bright field image of the tissues that were labeled with metal-conjugated antibodies and measured with LA-ICP-TOFMS. The figures below display two consecutive sections, with (B) representing a H&E stain and (C) a KI-67 antibody and DAB staining.

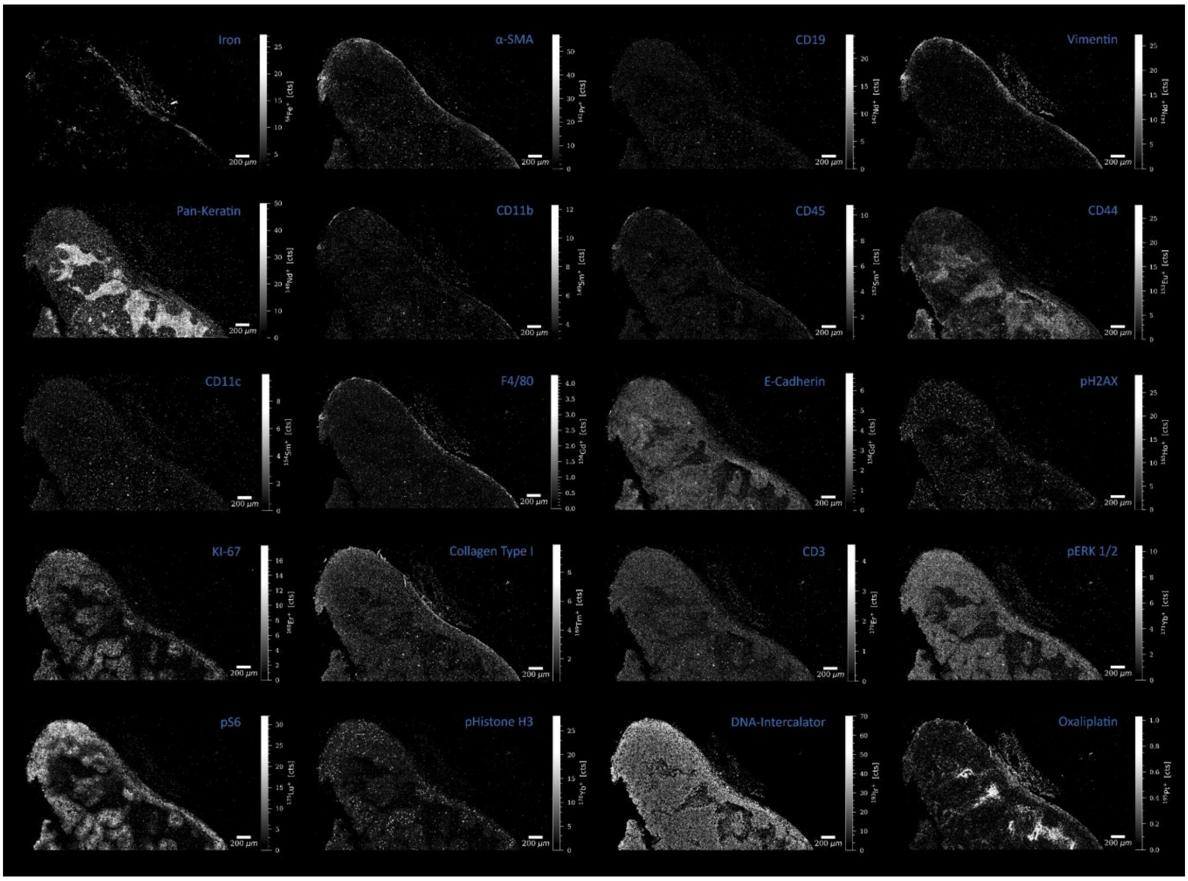

Figure S6: Signal intensity maps of all antibodies utilized in conjunction with both Fe and Pt for the HCT116/OxR tumor. Images were acquired using LA-ICP-TOFMS at a repetition rate of 300 Hz with a 1 μm pixel size.

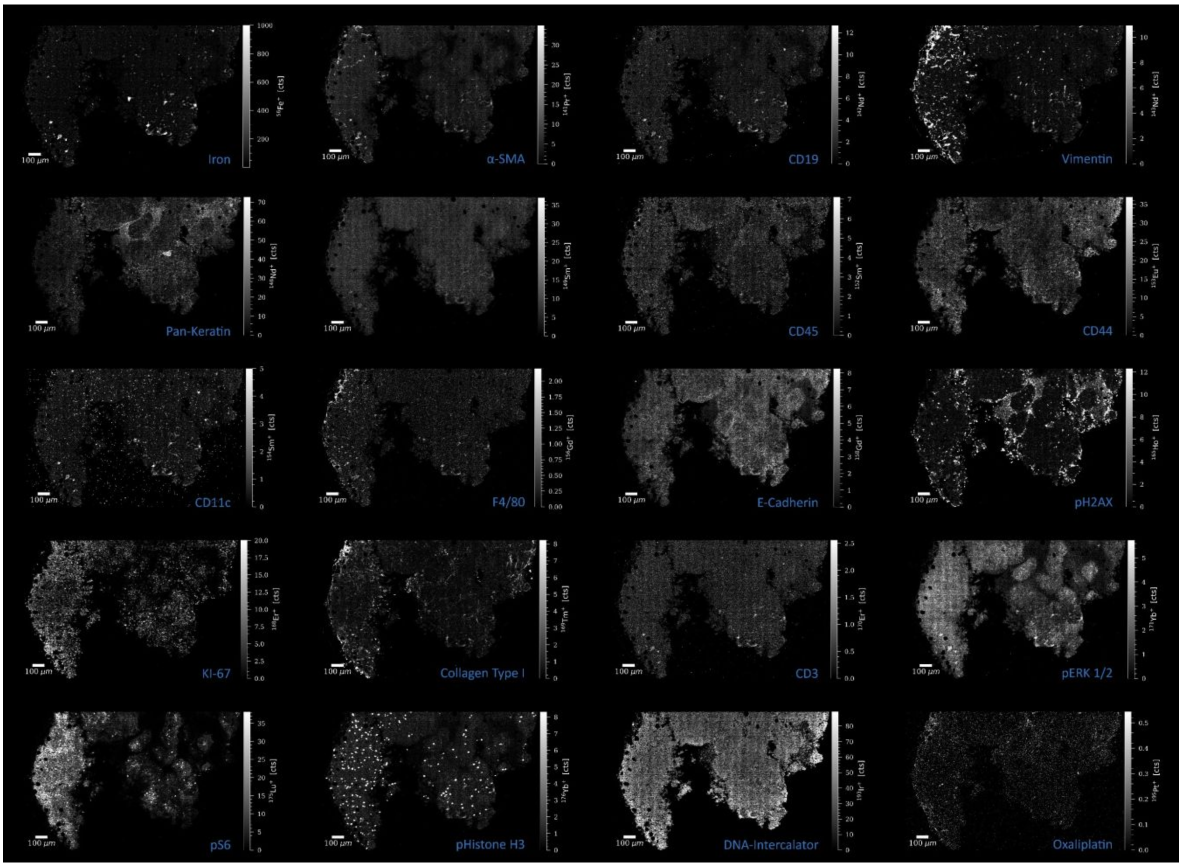

Figure S7: Signal intensity maps of all antibodies utilized in conjunction with both Fe and Pt for the parental HCT116 tumor. Images were acquired using LA-ICP-TOFMS at a repetition rate of 300 Hz with a 1 µm pixel size.

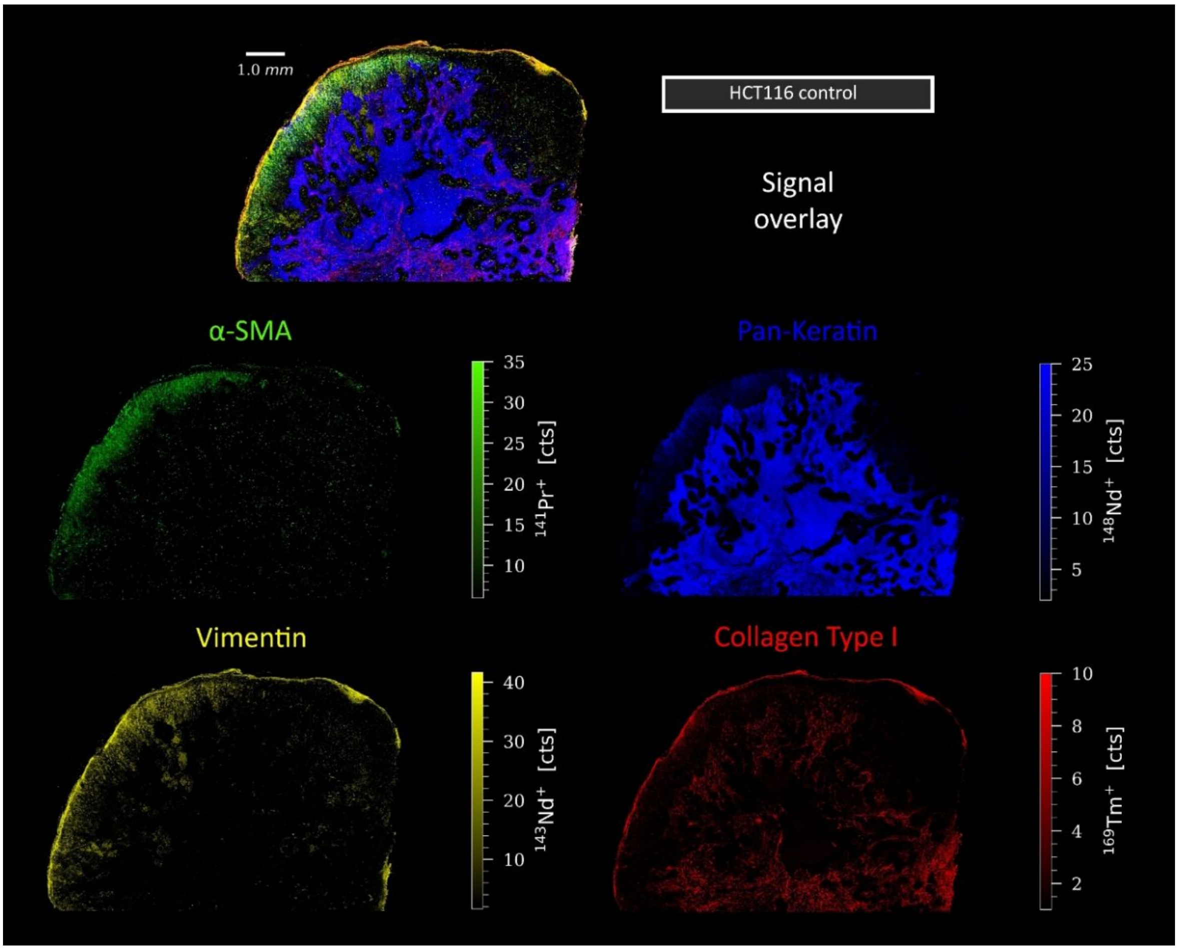

Figure S8: Structural characterization of a parental HCT116 tumor without OxPt treatment. The markers alpha-SMA, vimentin, pan-keratin, and collagen type I were employed. LA-ICP-TOFMS analysis was performed at a 300 Hz acquisition rate with a 5 µm pixel size due to the size of the tumor.

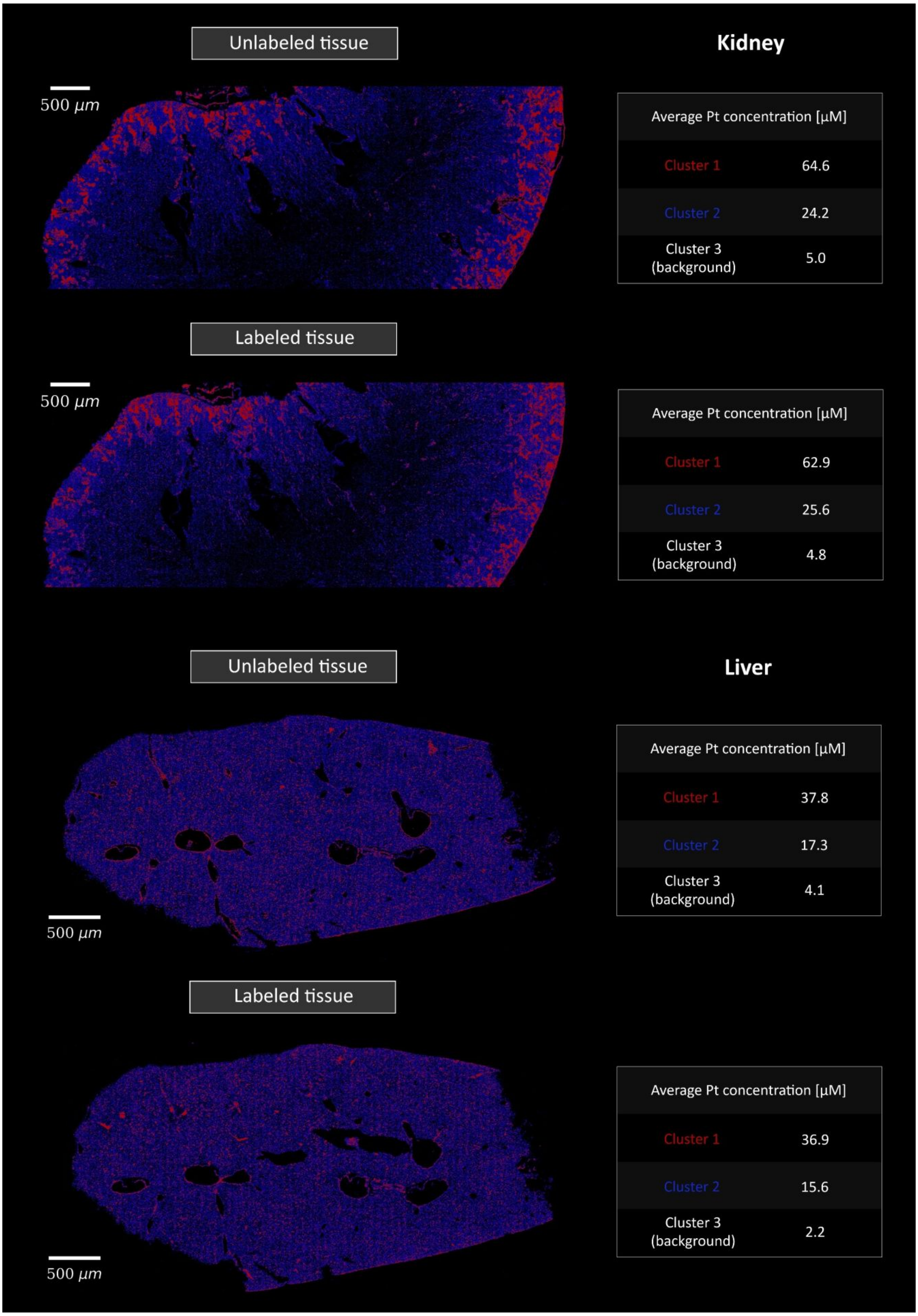

Figure S9: Pt distribution in consecutive sections of the kidney and liver, both unlabeled and labeled, along with the calculated average Pt concentrations from k-means clustering (n = 3).

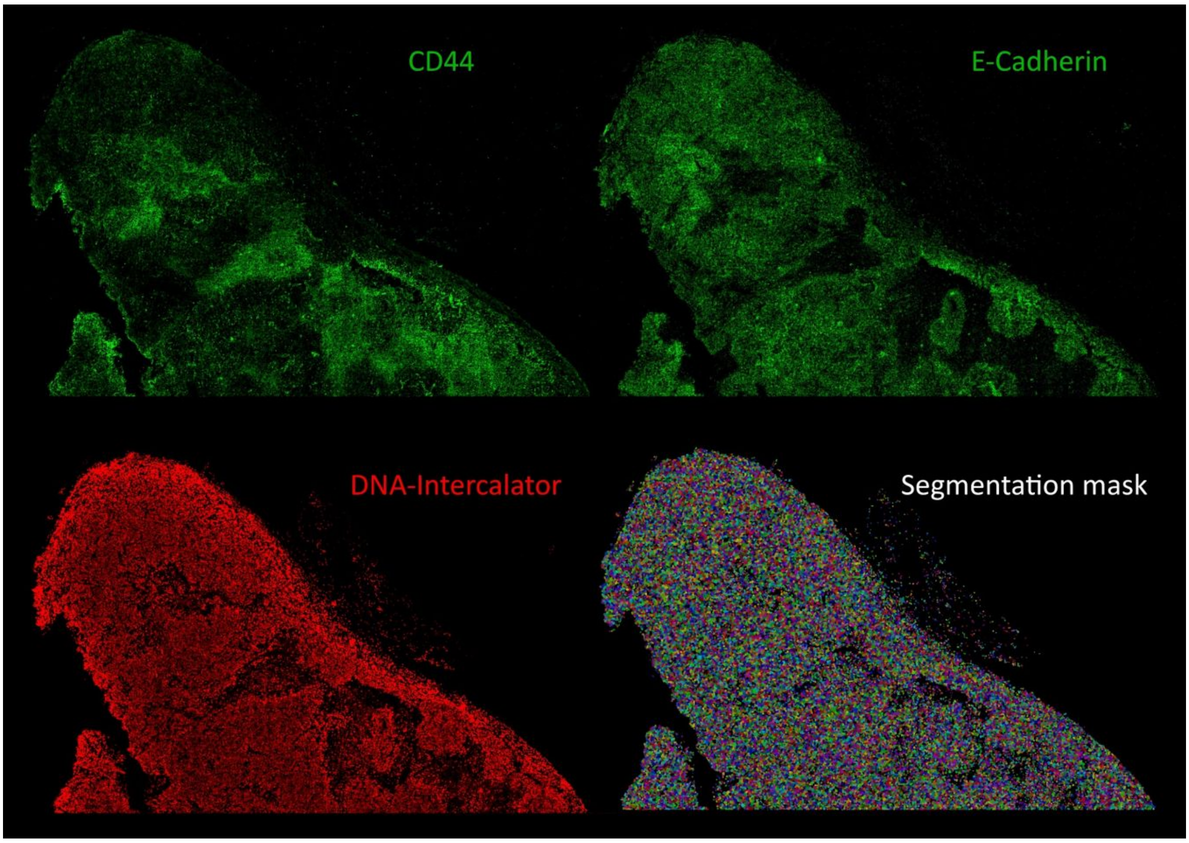

Figure S10: Given the distinct expression of CD44 and E-cadherin in the tissue, both were used as membrane markers to detect all cells. In combination with the signal produced by the DNA intercalator, a segmentation mask was made.

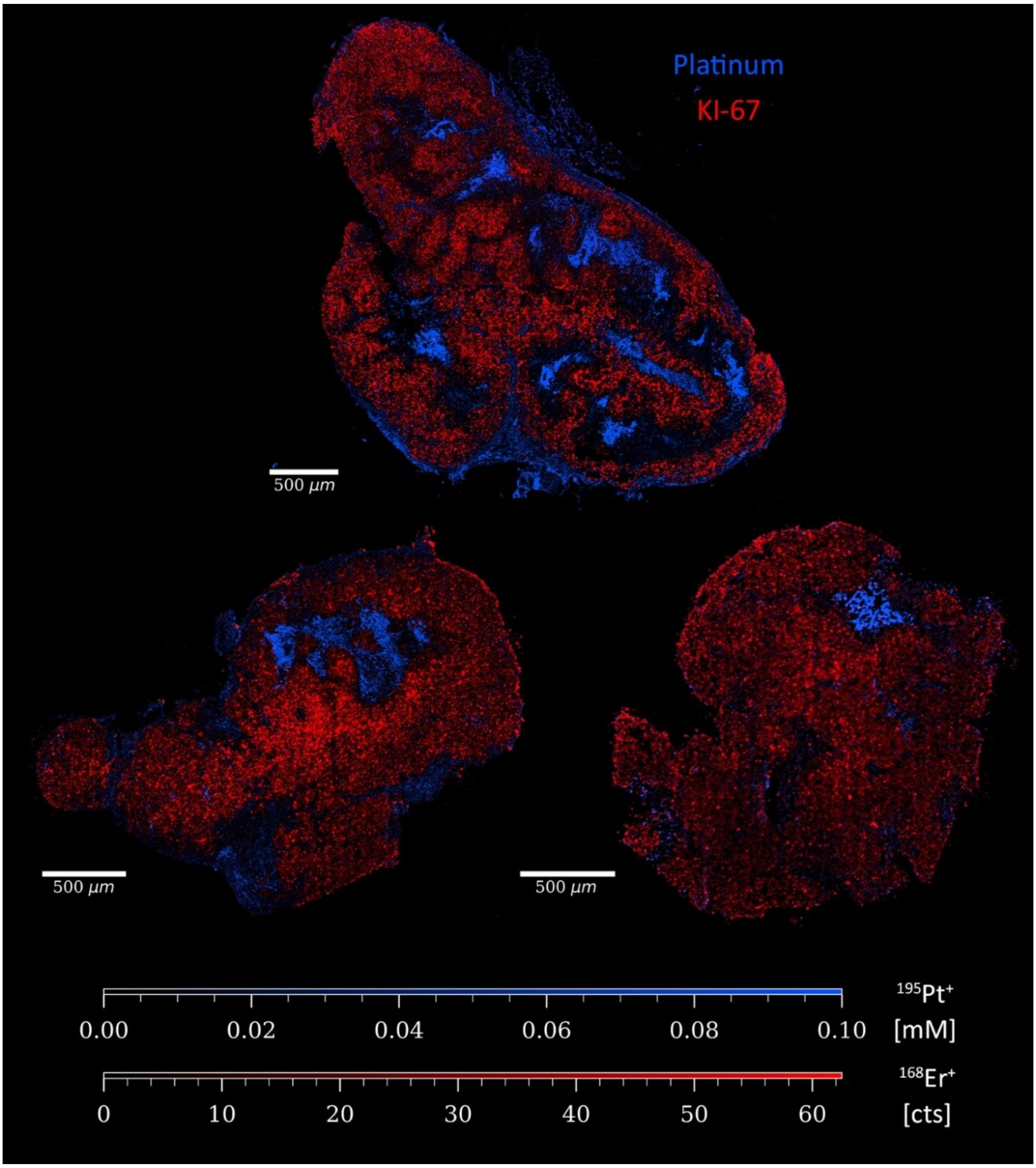

Figure S11: Overlay of Pt and KI-67 visualized for three different tumors of the resistant cell line. All tumors exhibited a significant Pt accumulation in necrotic regions, with comparable levels of Pt.

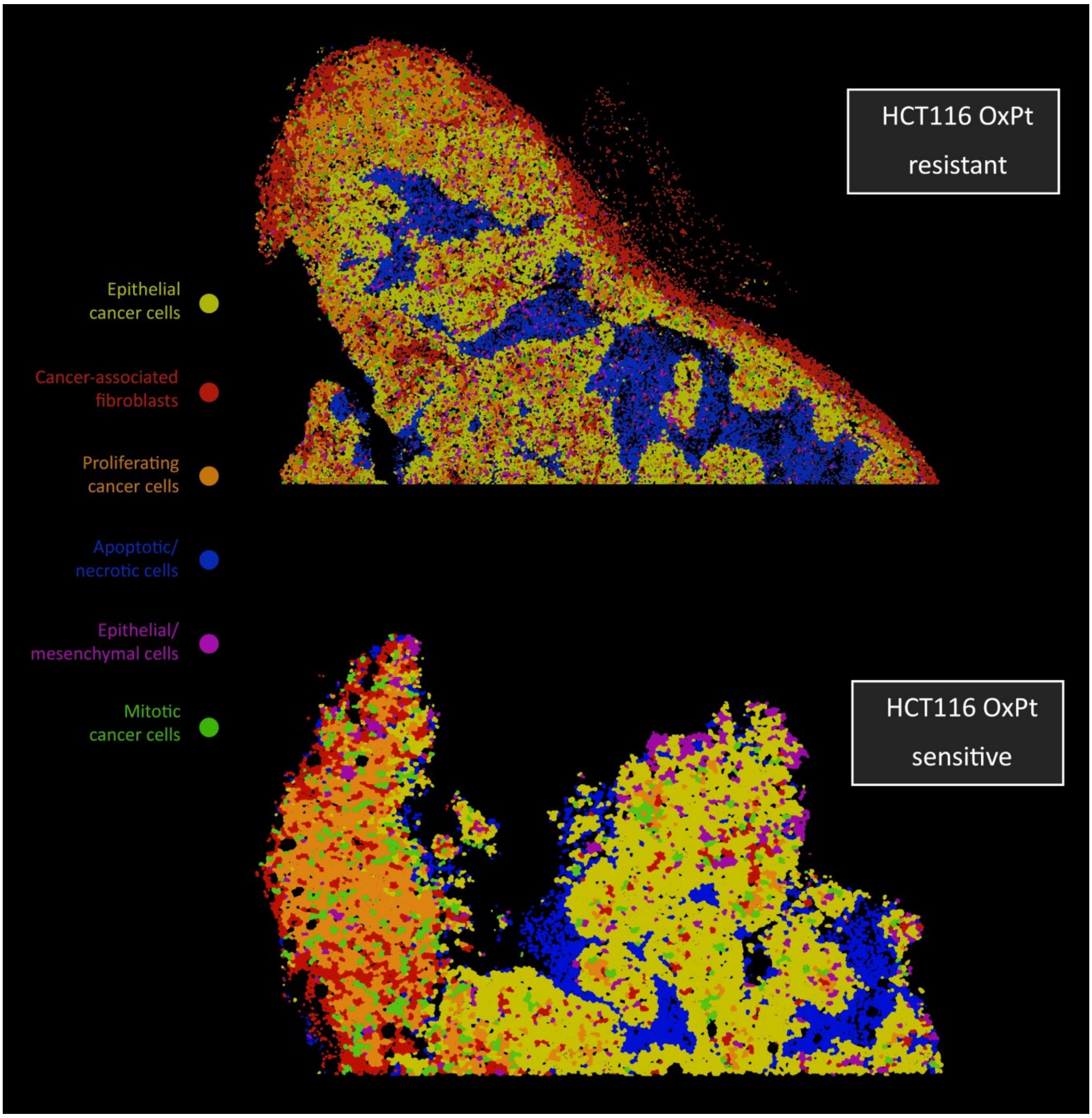

Figure S12: Visualization of the clusters generated by PhenoGraph clustering in the tumor tissues. The cellular phenotypes corresponded well with the tumor histology.

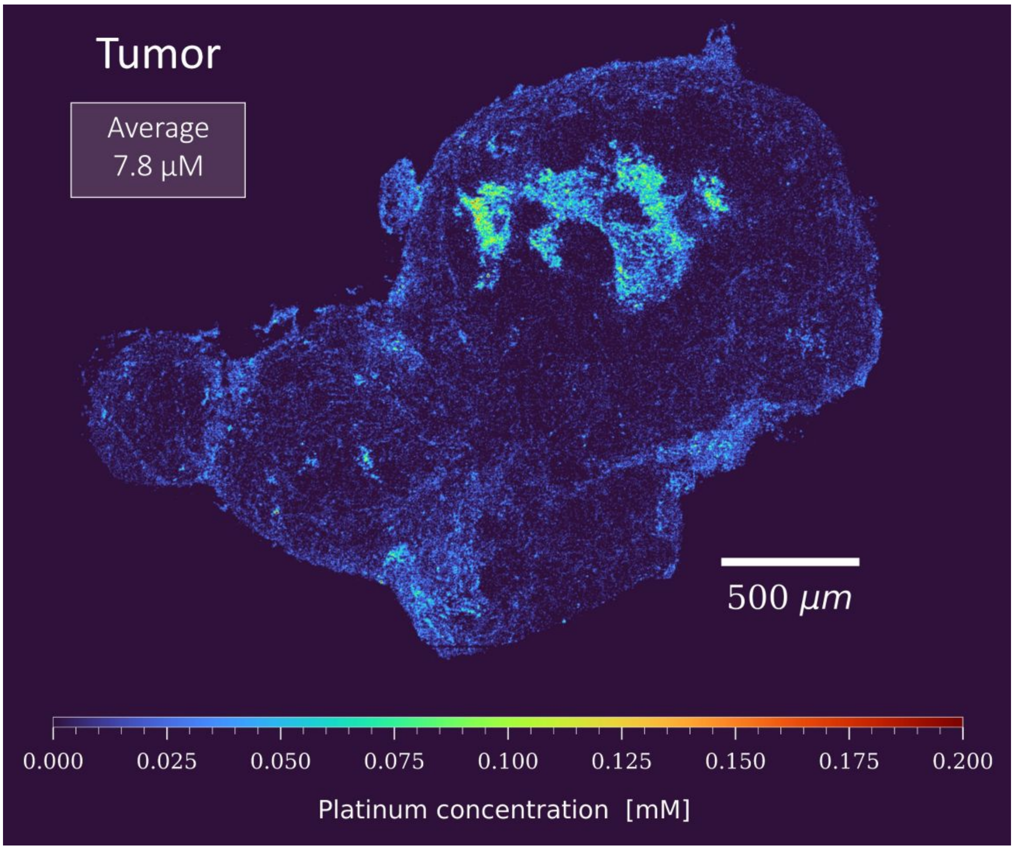

Figure S13: Quantitative Pt imaging in HCT116/OxR tumor section from an OxPt-treated mouse. The analysis was performed using LA-ICP-TOFMS imaging with a 300 Hz acquisition rate and 2.5 µm pixel size.

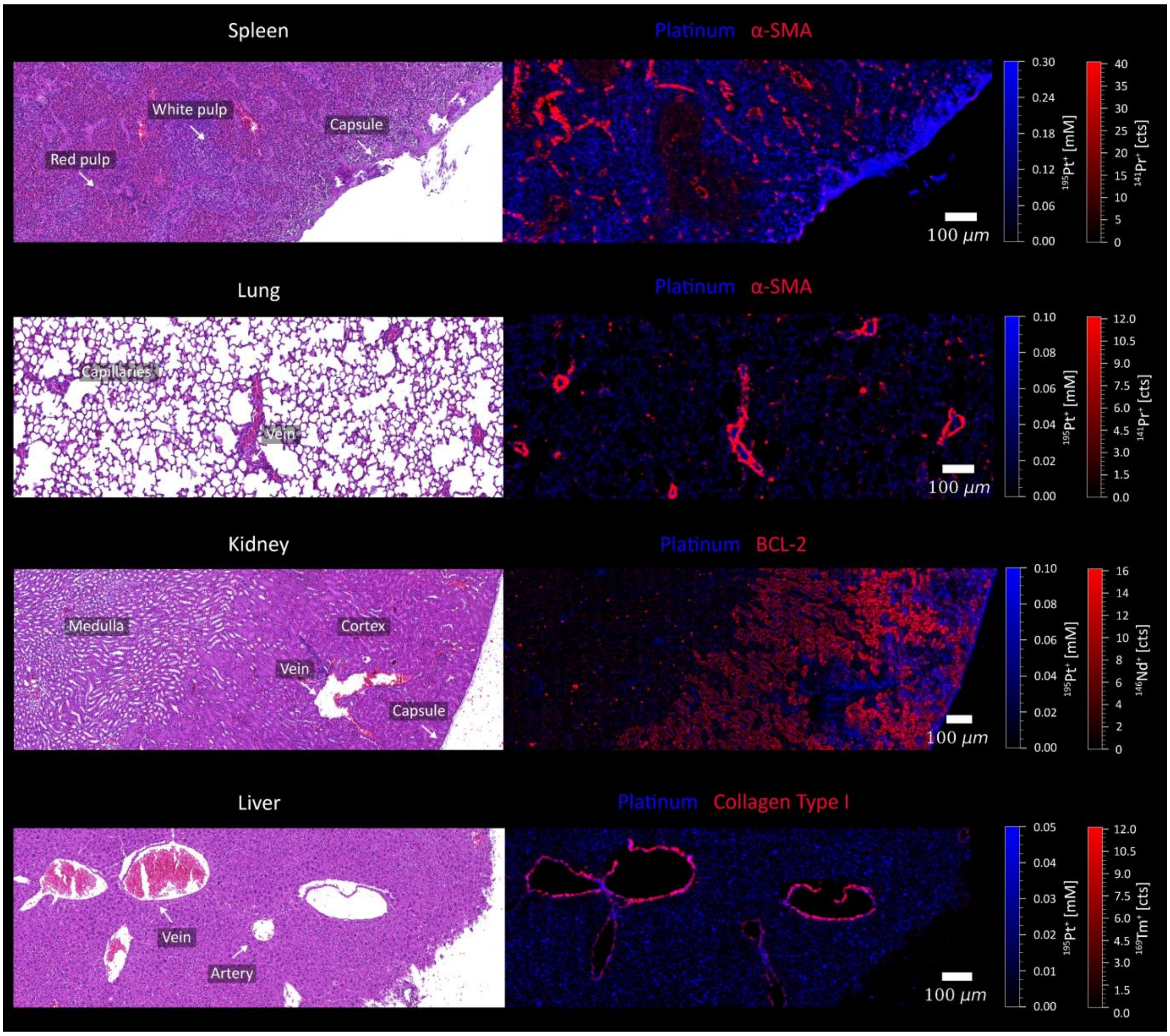

Figure S14: Combined imaging of Pt and metal-labeled antibodies in organ sections from an OxPt-treated mouse (right). The analysis was performed using LA-ICP-TOFMS imaging with a 300 Hz acquisition rate and 2.5 µm pixel size. Consecutive sections stained with H&E give an insight into the structural features of the organs (left).

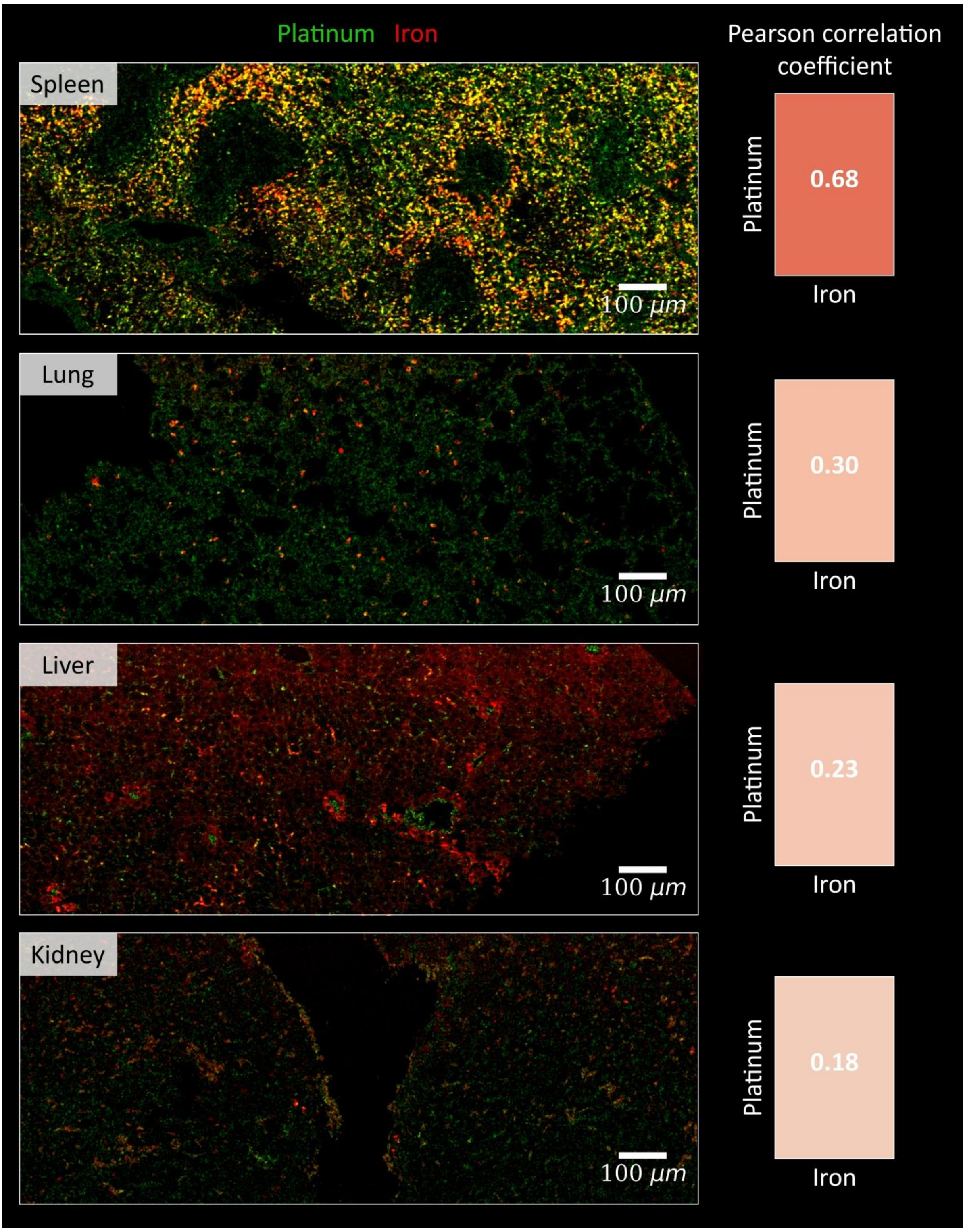

Figure S15: Imaging of Pt and Fe in OxPt-treated mouse organ sections, LA-ICP-TOFMS analysis, 1 µm pixel size, 300 Hz acquisition rate. An overlay of the signal intensity maps is shown in combination with the corresponding Pearson correlation coefficient. All organs showed a positive Pt/Fe correlation, with the spleen showing the strongest positive correlation at 0.68.

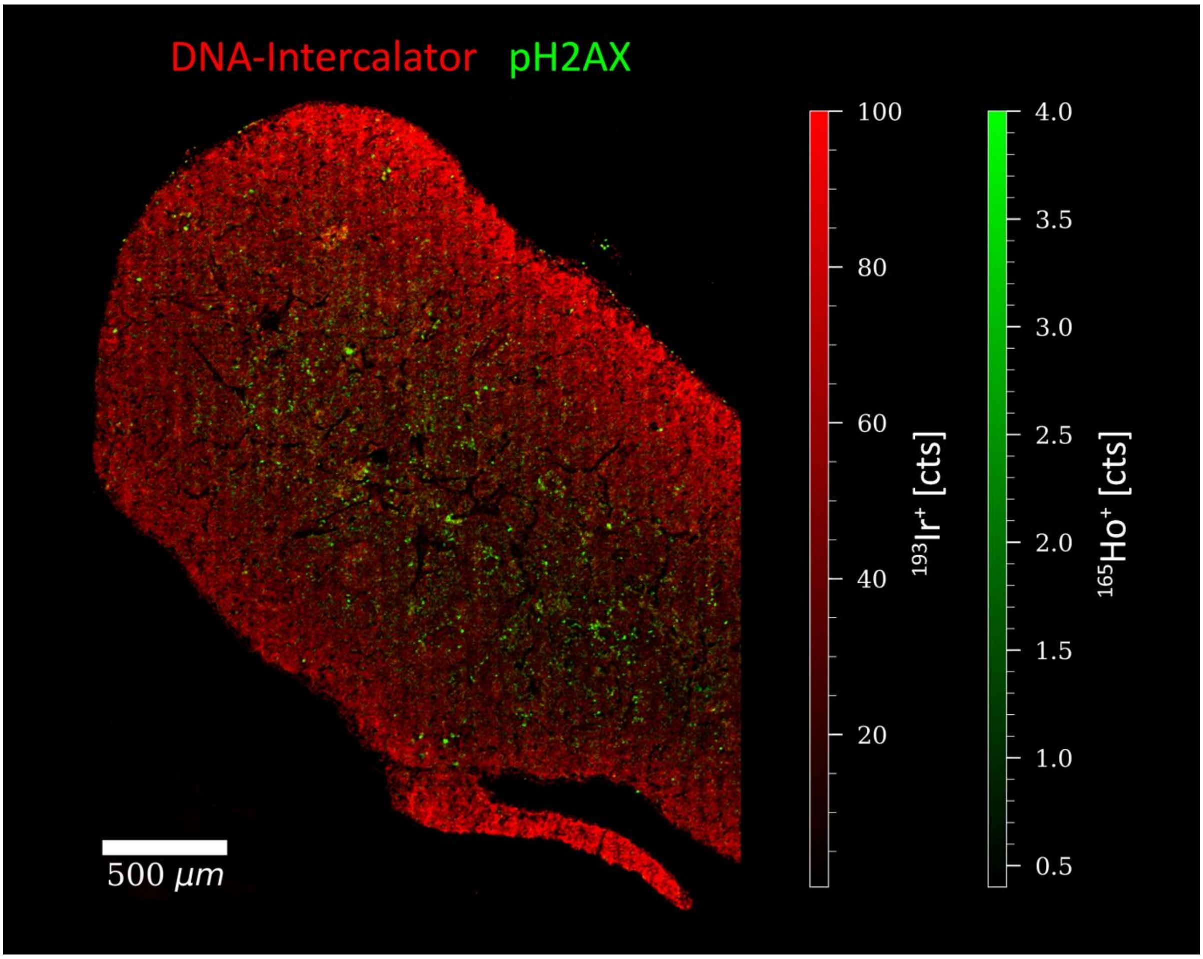

Figure S16: Imaging of DNA-Intercalator and pH2AX in OxPt-treated mouse spleen, LA-ICP-TOFMS analysis, 2.5 µm pixel size, 300 Hz acquisition rate. A considerable number of cells displayed evidence of DNA damage within the parenchyma.

Supplementary Tables S1 – S2

Table S1: List of metal-conjugated antibodies used for labeling of tumor tissue.

Antibody target	Clone	Metal tag	Function
**α-SMA**	1A4	^{141}Pr	Blood vessels/ myofibroblasts
**CD19**	6OMP31	^{142}Nd	B cells
**Vimentin**	D21H3	^{143}Nd	Mesenchymal cells/ fibroblasts
**Pan-Keratin**	C11	^{148}Nd	Epithelial cells
**CD45**	D3F8Q	^{152}Sm	Pan leucocyte
**CD44**	IM7	^{153}Eu	Tumor/EMT
**CD11c**	3.9	^{154}Sm	Dendritic cells
**F4/80**	D2S9R	^{156}Gd	Pan macrophage
**E-Cadherin**	24E10	^{158}Gd	Epithelial cell membrane
**pH2AX**	S139	^{165}Ho	DNA damage
**KI-67**	B56	^{168}Er	Proliferation
**Collagen Type I**	Polyclonal	^{169}Tm	Extracellular matrix
**CD3**	Polyclonal, C-Terminal	^{170}Er	Pan T cell
**pERK1/2**	T202/Y204	^{171}Yb	Apoptosis inhibition
**pS6**	S235/236	^{175}Lu	mTOR
**pHistone H3**	Ser28	^{176}Yb	Mitosis

Table S2: LA-ICP-TOFMS parameters.

**ICP-TOFMS**	
RF Power [W]	1440
Sampling depth [mm]	3.5
Cone materials	Ni
Plasma gas flow [L min^{-1}]	14
Auxiliary gas flow [L min^{-1}]	0.82
Nebulizer gas flow [L min^{-1}]	1.01
Measurement mode	Collision cell technology (CCT)
CCT gas	93% He *(v/v)*, 7% H_2 *(v/v)*
CCT gas flow [mL min^{-1}]	4.30
m/z range	14-256
Integration time [ms]	3
**Laser ablation**	
Spot size [µm]	2 (circular), 5-10 (square)
Interspacing [µm]	1-5
Repetition rate [Hz]	300
Dosage	2
Fluence [J cm^{-2}]	0.4-1.4
